# Force-transducing molecular ensembles at growing microtubule tips control mitotic spindle size

**DOI:** 10.1038/s41467-024-54123-2

**Published:** 2024-11-14

**Authors:** Lee-Ya Chu, Daniel Stedman, Julian Gannon, Susan Cox, Georgii Pobegalov, Maxim I. Molodtsov

**Affiliations:** 1 The Francis Crick Institute, London, United Kingdom; 2 King’s College London, London, UK; 3 Department of Physics and Astronomy, University College London, London, United Kingdom

**Keywords:** Biophysics, Mitotic spindle, Microtubules

## Abstract

Correct mitotic spindle size is required for accurate chromosome segregation during cell division. It is controlled by mechanical forces generated by molecular motors and non-motor proteins acting on spindle microtubules. However, how forces generated by individual proteins enable bipolar spindle organization is not well understood. Here, we develop tools to measure contributions of individual molecules to this force balance. We show that microtubule plus-end binding proteins act at microtubule tips synergistically with minus-end directed motors to produce a system that can generate both pushing and pulling forces. To generate pushing force, the system harnesses forces generated by the growing tips of microtubules providing unique contribution to the force balance distinct from all other motors that act in the mitotic spindle. Our results reveal that microtubules are essential force generators for establishing spindle size and pave the way for understanding how mechanical forces can be fine-tuned to control the fidelity of chromosome segregation.

## Abstract (web summary)

Forces that lead to stable bipolar organization of mitotic spindles are poorly understood. Here the authors show that growing interpolar microtubules make a unique contribution to the overall force balance, distinct from all other motors in the spindle.

## Introduction

During cell division, microtubules form bipolar spindles that ensure accurate chromosome segregation. Mitotic spindle forms when microtubule organization centers separate, converting monopolar organization of microtubules in interphase into the bipolar spindle in mitosis. Separation of the asters and stabilization of the correct spindle length are driven by motor and non-motor proteins that act in antiparallel microtubule overlaps forming between the poles with plus-end and minus-end directed motors antagonizing each other in the overall balance of forces^1–3^. Once the spindle is established, in metaphase and anaphase it is stabilized in by interactions between motors, microtubules and crosslinkers via mechanisms that are beginning to be understood^4–6^. However, earlier events and mechanisms underlying pushing force generation that lead to the aster separation and generation of stable bipolar metaphase spindle still remain unclear^7^.

HSET is a kinesin-14 family minus-end directed motor whose activities support pole focusing in mitotic spindles^8,9^ and required for establishing correct spindle size^10,11^. In vitro, HSET generates pulling forces that slide antiparallel microtubule bundles in the direction opposite to the plus-end directed motors^12,13^, which is partially mediated by the HSET tail domains that interacts with microtubules diffusively^14,15^. This interaction also allows HSET to slide antiparallel microtubules with speeds that depend on both motor density and the size of the microtubule overlap^16^. Activity of HSET can generate asters and microtubule networks^17^ and affects spindle size^18^. However, HSET does not directly counteract plus-end motor activity that could result in efficient force balance leading to bipolar configuration^14,19^. To the contrary, depletion of HSET causes shorter, not longer, spindles as would be expected from the contribution of a minus-end directed motor to the overall force balance^10,20–22^.

HSET ability to shift force balance in mitotic spindles depends on the EB family of microtubule plus-tip tracking proteins^11,23,24^. EBs autonomously recognize growing microtubule tips and act as both regulators of microtubule dynamics as well as hubs that recruit large number of other regulatory proteins to growing microtubule tips^25^. EB depletion in mitosis is similar in phenotype to HSET. It causes shorter spindles^26,27^ and leads to misplaced and disorganized spindles as well as chromosome alignment defects resulting in chromosome segregation errors^28,29^. One mechanism how EBs and HSET affect spindle force balance is by HSET removing EBs from the tips of individual microtubules leading to the change in microtubule dynamics^11^. However, whether EB and HSET can directly participate in the force generation acting at the tips of antiparallel spindle microtubule overlaps is unknown.

Not only molecular motors but also microtubules themselves can generate mechanical force. Growing microtubule tips can push against physical obstacles generating forces of up to 5 pN^30^, which implicates them in cellular processes where microtubules push against physical surfaces such as membranes^31–37^. Growing microtubule tips are abundant in the spindle, raising possibility that they could be important force generators contributing to the spindle force balance. However, microtubules cannot push directly on other microtubules or microtubule asters by the same mechanism they use to push against microfabricated barriers, plastic beads or membranes^38^. Applying forces to other molecules must require presence of transducers that could harness forces generated by microtubule growth and convert them into mechanical work in the mitotic spindle. Likely candidate for such role could be microtubule tip trackers such as EBs, but initial studies showed that individual EB proteins are not efficient force couplers^36^.

EBs can form multivalent interactions at microtubule tips with various partners^39^. EB1 and EB3 interactions with HSET can reverse the minus-end directed movement of the motors and change the direction of microtubule growth suggesting they can generate force^24^. Studies using other proteins capable of harnessing microtubule generated forces showed that multivalent interactions between microtubule binding proteins is an efficient way of harnessing forces generated by microtubule dynamics^40–43^. This suggests that multivalent interactions between EBs at the tips of spindle microtubule could contribute to force generation. However, whether combination of HSET motors and EB tip trackers can efficiently harness forces generated by microtubule growth and whether this force can be substantial contributor to the force balance in mitotic spindle in unknown.

## Results

### A single minus-end directed motor, dynamic microtubules and tip trackers generate stable bipolar organization

**Fig. 1:**
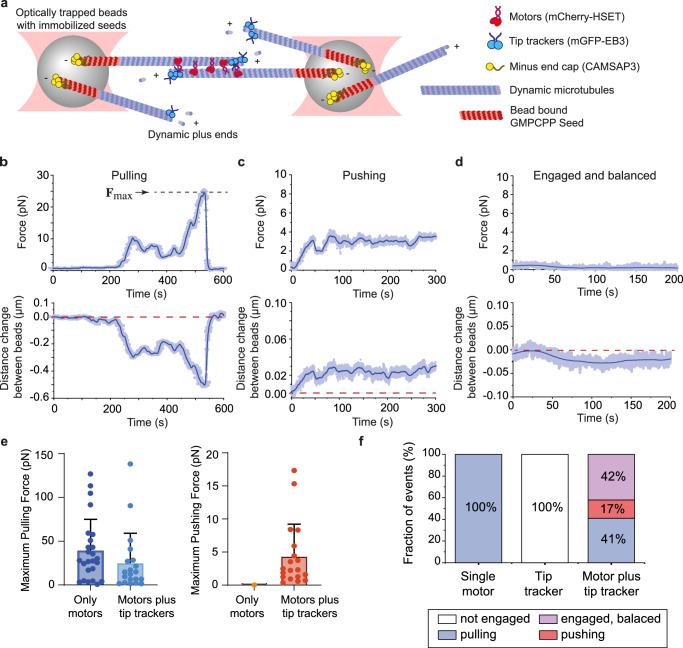
Microtubule dynamics, molecular motors and tip trackers stabilize bipolar organization of two microtubule asters. **a** Schematics of the double optical trap artificial spindle assay. Microtubule seeds are tethered to plastic beads that are held in two optical traps and capped at their minus ends to mimic two microtubule organization centers. The assay measures the force acting between asters. **b** Example recording shows pulling force (top) and the corresponding decrease in distance between the beads (bottom) in the presence of HSET only. Maximum force inferred from traces is shown. **c** Example recording shows pushing force (top) and the increase in the distance between beads (bottom) in the presence of EB3 and HSET. **d** Example recording of a balance trace with no force generated while microtubules are engaged in the presence of EB3 and HSET. In **b**–**d** light blue are original data and dark blue is smoothed with 100 point moving average. **e** Histograms of maximum pulling and pushing forces extracted from individual traces. Bars show mean values and whiskers standard deviation. Number of measurements (left to right): *n* = 24, *n* = 20, *n* = 0 (no events detected), *n* = 19. **f** Quantification of the fraction of time that the system spends pushing, pulling, and being balanced (with at least two antiparallel microtubules from the opposite asters engaged) or disengaged (when microtubules from opposite asters do not interact) depending on the experimental conditions (*n* = 84 total traces). Source data for this figure are provided as a Source Data file.

To address this question, we established an artificial spindle assay which allowed measuring forces exerted by microtubules on the spindle poles in vitro. In this assay, dynamic microtubule plus ends were grown from stabilized microtubule seeds with their minus ends blocked and immobilized on two optically trapped beads, mimicking the aster organization at the microtubule organizing centers (Fig. 1a).

First, we supplemented the reaction with a minus-end directed motor HSET, which is known to crosslink and slide antiparallel microtubules^12,13^. As expected for a minus-end motor, as soon as microtubules from the opposite asters engaged, distance between the beads decreased due to the pulling force generated by the motor (Fig. 1b, Supplementary Movie 1). Increase in the force that we measured by the optical trap was typically followed by an abrupt drop, which was either due to one of the engaged microtubules breaking or undergoing a catastrophe. The drop was frequently followed by another force increase due to engagement between a different pair of other microtubules, which resulted in a saw-tooth like force pattern (more examples are in the Supplementary Fig. 1a–c).

Next, we added microtubule tip tracking protein EB3, which is known to bind HSET such that it makes microtubule binding domains on both EB3 and HSET available for engaging microtubules^11,44^, after having verified their interaction (Supplementary Fig. 1d, Supplementary Movie 2). Although EB3 is not a motor, when both minus end directed motor and the tip tracker were present in our assay, we observed not only pulling, but also pushing as was indicated by the increase in the distance between the beads (Fig. 1c–e and Supplementary Fig. 1, Supplementary Movie 3). The switching between pulling and pushing appeared to be stochastic (Supplementary Fig. 1e), and our analysis revealed that ~40% of the time microtubules growing from opposite beads were engaged in the presence of EB3 and HSET, but no overall force was detected indicating that pushing and pulling forces were balanced and the system was in a stable bipolar state (Fig. 1f, Supplementary Fig. 1b). With EBs alone, microtubules did not engage, and we could not detect any force between microtubule asters (Supplementary Fig. 1c). These data suggested generation of pushing forces in antiparallel microtubule networks depended on the combination of the minus-end directed motor and a tip tracker. Intriguingly, our data suggested that a simple system consisting of a single molecular motor and dynamic microtubules with tip trackers could produce a stable bipolar state.

### Two growing antiparallel microtubules push with up to 5 pN

**Fig. 2:**
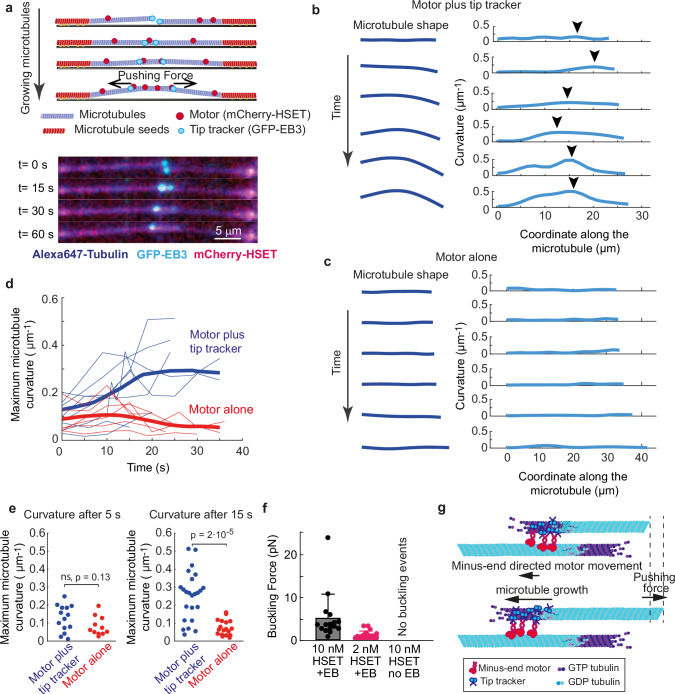
Two antiparallel growing microtubules generate pushing force. **a** Schematics and montage images of an example buckling event between two antiparallel microtubules (See also Supplementary Fig. 2a). At least 10 independent experiments were carried out. **b** Shapes of a microtubule undergoing buckling in the presence of 100 nM EB3 and 10 nM HSET as it grows in an antiparallel overlap. Only one microtubule from pair is shown. On the right - curvatures of the microtubule at the corresponding time points along the microtubule length. Arrowhead points to the maximum curvature. **c** Same as in ‘b’, but no EB3 present (HSET only). **d** Maximum microtubule curvatures determined from ‘**b**’ and ‘**c**’ plotted as function of time passed since the first antiparallel engagement event. Thin curves are individual examples and thick curves are averages. Blue corresponds to 100 nM EB3 and 10 nM HSET, red − 10 nM HSET. **e** Curvatures of all microtubules digitized following 5 and 15 seconds after engagement is shown. Significance calculated by the two-sided Wilcoxon test. **f** Buckling force measured for different HSET concentrations. Bars are mean and whiskers standard deviation. Number of measurements (left to right): *n* = 16, *n* = 19, *n* = 0 (zero events detected). **g** Schematics of the model proposing how coupling between minus-end directed motor and a growing microtubule tip can harness pushing force generated by the microtubule growth. Protein action is shown at one microtubule tip only. HSET complexes acting in the overlap as well as HSET and EB acting at the other tips are not shown for simplicity. Source data for this figure are provided as a Source Data file.

The maximum pushing forces that we measured in our optical trapping assay rarely exceeded 5 pN (Fig. 1e). Since most of the load on the beads was likely carried out by a single pair of the antiparallel microtubules, we reasoned that this presumably corresponded to the maximum force the pair can generate. However, the exact number of engaged microtubules was poorly controlled in the optical trapping assay. To measure the force generated by our system in a single pair of two antiparallel microtubules, we used an alternative assay in which we attached stabilized microtubule seeds to the surface of a passivated coverslip. Out of all microtubules that grew from randomly oriented seeds, we selected pairs that had their plus-ends in approximately antiparallel configuration (Fig. 2a, *n* = 25 total engagement events selected). After the encounter, the tips engaged in the presence of HSET, and continued to grow along one another with HSET enriched in the antiparallel overlap as expected^16^ (Supplementary Fig. 2a). Notably, however, in the presence of both motor and tip tracker, microtubules that continued to grow into antiparallel overlaps started to buckle (Fig. 2a, Supplementary Movie 4), and became visibly bent (Fig. 2b).

Buckling indicates compressive force acting along microtubules which can only be explained by the microtubule tips pushing against each other. To quantify this, we traced the shapes of microtubules, calculated their curvatures, and plotted the maximum curvature of microtubules as a function of time after it first engages in an antiparallel overlap (Fig. 2b–d). This analysis confirmed that both EB3 and HSET were required to generate pushing force and no detectable buckling, and therefore pushing, was observed with the motor alone (Fig. 2e).

To explore whether interaction between EB3, HSET and microtubules in antiparallel overlaps stabilized EB/HSET complexes, we temporary increased imaging laser power ~10-fold to photobleach both EB3 and HSET. We then monitored fluorescence recovery of both complexes at the free microtubule tips and in antiparallel overlaps. On single microtubule tips and in the presence of HSET, individual EB3 recovered within ~6 s (Supplementary Fig. 2b, c). However, on the tips in antiparallel overlaps turnover of EB3 slowed down almost threefold and recovered at the same rate as HSET (Supplementary Fig. 2d, e) consistent with the idea that both proteins acted together at the tips of microtubules forming antiparallel overlaps. However, the rate of microtubule growth did not change after individual microtubules engaged in pushing force generation in the antiparallel overlaps (Supplementary Fig. 2f).

To infer the magnitudes of pushing forces, we extracted microtubule lengths at the initial moment of buckling from their images and converted them into force using the relationship between the flexural rigidity of microtubules, their length and buckling^30,45^ (Supplementary Fig. 2g). At 2 nM HSET, we only observed buckling of long microtubules, which when converted into buckling force corresponded to forces of approximately 1 pN. At 10 nM HSET multiple shorter microtubules buckled, indicating generation of higher pushing forces (Fig. 2f). The distribution of the pushing forces was highly non-normal (*p*-value $4\cdot {10}^{-13}$, Kolmogorov-Smirnov test) and decreased abruptly at forces above 5 pN, suggesting that this is approximately the maximum force that the system can develop. These measurements were consistent with the forces measured in the optical trapping assay and reinforced our conclusion that EB/HSET system can generate up to ~5 pN pushing force in a single pair of growing antiparallel microtubules.

### EB3 dimers connected by a flexible linker move processively with microtubule tips

Next, we sought to understand how tip tracking proteins contribute to the generation of pushing forces in antiparallel microtubule bundles. Since individual EBs bind microtubule tips only transiently, the mechanism likely depends on the combined action of multiple EBs at the microtubule tip that are connected to HSET. In dividing cells, the rate of the microtubule growth is larger than the speed of HSET movement^27,46^, which was also true in our in vitro experiments (Supplementary Fig. 3a). We reasoned, that if multiple EBs can bind and unbind from the microtubule tip while staying connected to the HSET moving on the antiparallel microtubule, they could possibly couple the growth of the microtubule tip to the minus-end movement of HSET along the antiparallel microtubule (Fig. 2g). Since microtubule grows faster than motor proteins move, the coupling between the two would result in the overall increase of the antiparallel bundle length, which would translate into the generation of the pushing force.

**Fig. 3:**
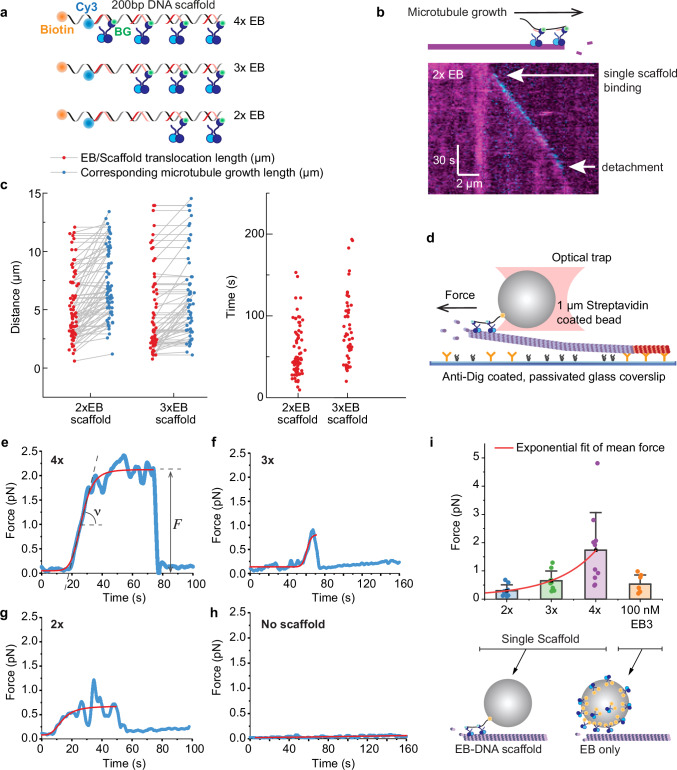
EB dimers connected by a flexible scaffold move processively and harness force from a growing microtubule tip. **a** Schematics of EB3 ensembles on a 200 bp DNA scaffold. **b** Kymograph of a single DNA scaffold with two EB3 heterodimers tracking the tip of a growing microtubule. **c** (Left), Red points are run lengths of individual EB3 scaffolds. Blue points are growth distances of the microtubule tips that these scaffolds ran on. Corresponding tips and scaffolds are connected by lines. (Right) residency times of the scaffolds from the left (*n* = 162 for 2xEB3, *n* = 56 for 3xEB3). **d** Schematics of the experimental setup for measuring forces generated by EB3 scaffolds. **e**–**h** Individual examples of force traces for scaffolds with different numbers of EBs. Traces shown are smoothed to 20 Hz and fitted with logistic function (red line) to determine the force. In ‘**e**’ extracted force and slope are shown ($F$ and $\nu$). 42 out of 200 beads tested showed detectable force generation. **i** Extracted forces plotted as a function of the number of EBs in the scaffold. Bars are mean and whiskers are standard deviation. Number of measurements left to right: *n* = 9, *n* = 9, *n* = 10, *n* = 7. On the right, experiment in which EB dimers were coupled directly to the streptavidin bead (No scaffold used. One bead has estimated > 170,000 EB binding sites). In ‘**e**–**i**’ 2x, 3x and 4x corresponds to single scaffolds with 2, 3 and 4 EB3 dimers respectively. Source data for this figure are provided as a Source Data file.

This suggested that several EBs connected to an appropriate mechanical scaffold could form a coupling device capable of harnessing up to 5 pN force from a single microtubule. To test this directly, we generated ensembles containing exactly 1, 2, 3 and 4 full length EB3 dimers covalently connected via a single SNAP-tag to an artificial scaffold that we made from DNA with a single Cy3 fluorophore (Fig. 3a, Supplementary Fig. 3a, b). At 1 nM concentration, single scaffolds containing just two EB3 dimers (that are not motors themselves) showed processive movement driven by the growing microtubule tip (Fig. 3b). We could not detect any binding of scaffolds containing just one EB3 dimer or no EBs at all to the growing microtubule tips under these conditions, and the quantification of fluorescence confirmed that we indeed observed processive movement of single scaffolds with two EB3 dimers (Supplementary Fig. 3d–g).

Scaffolds with two EB3 dimers tracked microtubule tips for up to several microns and stayed engaged for up to 100 s (Fig. 3c). When we increased the number of dimers to three, the run length did not substantially increase because in both cases it was limited by the extent of the microtubule growth (Fig. 3c). This shows that unlike individual EB3 dimers, which are non-processive and turn over quickly at microtubule tips, EB3 dimers connected by flexible linkers work as an efficient coupling device that harnesses growth of the microtubule tips to produce reliable and processive movement.

### EB3 harness several piconewtons of force from single growing microtubule tips

Next, we asked how much force can be harnessed from the growing microtubule tip by the EB-DNA scaffold. We attached DNA scaffold via biotin to a streptavidin-coated plastic bead, trapped the bead in a single-beam optical trap, positioned it in front of the growing microtubule and recorded the mechanical transients associated with the bead movement caused by its interaction with the growing microtubule tip. We ensured that the interaction between the bead and the microtubule tip was mediated by a single scaffold (Fig. 3d, see “Methods”).

When a scaffold driven by the microtubule growth exerted force on the bead, the force increased, plateaued, and then dropped back to zero after the scaffold detached from the microtubule (Fig. 3e–h). We counted only those signals in which the force increased approximately with the speed of the microtubule growth when the tip reached the bead. With only one EB3 heterodimer per scaffold, we failed to detect any signals satisfying these criteria. With two EB3 heterodimers per scaffold the detection of force generation became robust with 9 out of 50 traces showing the increase in force with the speed of microtubule growth and the maximum force of ~1 pN (Fig. 3i).

The maximum force generated by the scaffold increased approximately exponentially with the number of EB3 dimers in it (Fig. 3i). The maximum number of EB3 dimers per scaffold that we could test was four, but even with this amount, forces frequently reached above 2 pN, which is a significant contribution to the maximum force of 5 pN that a single microtubule tip generated (see in Fig. 1 and Fig. 2). Assuming the exponential dependence inferred from our data, we extrapolate that possibly as little as five to eight EB3 dimers could already be sufficient to harness pushing forces up to 5 pN from a growing microtubule tip. In a control experiment, we coated streptavidin beads directly with saturating amount of biotinylated EB3 dimers. In this case, up to a hundred of thousands of EBs are expected to be attached to the bead, but we only detected forces below 1 pN (Fig. 3i, 0.5 ± 0.3 pN mean ± SD), consistent with previous measurements^36^. Thus, unlike individual dimers, EBs connected by a flexible scaffold form an efficient coupler that can harness mechanical force from growing microtubule tips.

### Microtubule/EB/HSET system establishes bipolar microtubule organization

**Fig. 4:**
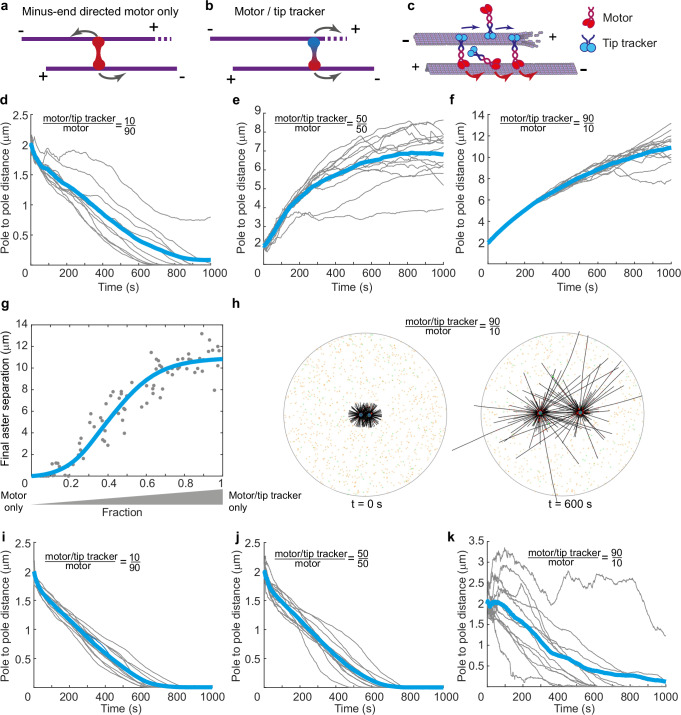
Organization of the two microtubule asters by the microtubule/motor/tip tracker system. **a**, **b** Schematics of the activity of minus-end directed motors and their combination with tip tracker in antiparallel microtubule overlaps. **c** In the model one motor/tip tracker unit is represented as EB scaffold in which EBs are connected via HSET to the antiparallel microtubule. **d**–**f** Pole-to-pole distances as a function of time for different ratios between motors alone and motor/tip tracker complexes. Gray lines are individual simulations, blue lines are averages. Microtubule growth was 2x faster than motor movement (Supplementary Fig. 2b). **g** Steady state pole-to-pole separation as a fraction of the motors/tip tracker complexes to all complexes in the system. Line is trendline. **h** Snapshot images of the simulations for the 90% motor/tip tracker complexes in the beginning and in the middle of the simulation (Corresponds to Supplementary Movie 6). Colors: Black – microtubules, Green – unbound HSET, Orange – unbound EB/HSET, Dark green – microtubule bound HSET, Red – microtubule bound EB/HSET. **i**–**k** Pole-to-pole distances as a function of time in the simulations for different ratios between HSET and EB/HSET in which EB/HSET was assumed to detach at forces exceeding 0.1 pN. Notations follow (**d**–**f**). Source data for this figure are provided as a Source Data file.

To gain further insight into the force-generating properties of the EB/HSET system and understand how it could facilitate stabilization of the bipolar configuration, we simulated the interaction between two 2D asters of microtubules in the presence of minus-end directed motors and their complexes with microtubule tip trackers using Cytosim^47^ (Methods, Fig. 4a–c). As expected, when the interaction between microtubules was dominated by the minus-end directed motor alone, two microtubule asters rapidly fused (Fig. 4d, Supplementary Movie 5). However, when the fraction of the motor in the complex with EBs was increased, distance between asters increased until it plateaued leading to a stable bipolar configuration (Fig. 4e, f, Supplementary Movie 6). This illustrates that the combination of dynamic microtubules, a minus-end directed motor, and a plus-end tracker provides a minimal system that can separate two asters of microtubules and provide stable steady-state distance in a bipolar configuration. Our simulations revealed that the steady-state distance between the asters did not depend on the absolute number of the molecules but depended on the microtubule dynamic instability parameters (Supplementary Fig. 4a, b).

In this simplified model we represented tip tracking as a collective action of multiple EBs by assuming that the motor/tip tracking complexes at microtubule tips generate up to 5 pN force as was suggested by our experiments. To test whether ability of tip tracking complexes to generate force was essential for the stabilization of the bipolar organization we compared these simulations to the simulations in which we assumed that EBs cannot transmit force from microtubule tips and detach at forces exceeding 0.1 pN as would be expected for individual unconnected EB dimers. These simulations revealed that at any of the motor/tip tracker ratios microtubule asters fused and could not maintain bipolar configuration (Fig. 4i–k). The limitation of these simulations is that they do not have all the components of the mitotic spindle assembly. However, they qualitatively illustrate how the ability of EBs to harness forces generated by microtubule growth can enable the EB/HSET system to generate pushing forces between the microtubule asters and facilitate the establishment of the bipolar state.

### Pushing forces generated by antiparallel microtubules are required for correct spindle size in human cells

**Fig. 5:**
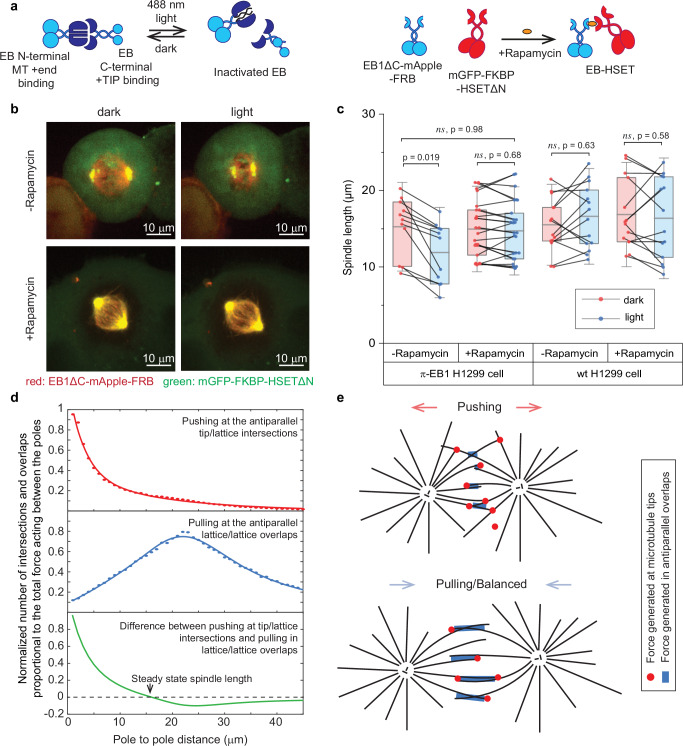
Metaphase spindle length in H1299 cells depends on the pushing force generated by EB/HSET. **a** Schematics of the two systems that allow endogenous EB light inactivation (π-EB1) and rapamycin-induced EB3 – HSET interaction. **b** Fluorescent images of π-EB1 H1299 cells with or without rapamycin treatment, light and dark blue-light activation. EB1 light inactivation induces spindle shortening, but only in cells lacking additional rapamycin activated EB/HSET system. At least five independent experiments with the same conditions independently for π-EB1 H1299 cells and wt H1299 cells were carried out. **c** Spindle length measured in H1299 and π-EB1 H1299 cells before (red points) and after (blue points) EB light deactivation with and without rapamycin. Gray lines connect single cell spindle length measurements before and after the blue light exposure. The boxes show mean, lower and upper quartiles. Whiskers represent ranges that fall within 1.5 times the interquartile range. Individual independent measurements are also shown. Number of measurements left to right: *n* = 11, 11, 26, 26, 14, 14, 14, 14. Significance was tested by Wilcoxon two-sided test, exact p values are shown. **d** Top data are simulations of the number of antiparallel tip/lattice interactions (red) and the area of the antiparallel lattice/lattice overlaps (blue) as the function of the pole-pole distance between asters. Points are simulations, lines are trendlines. Green is the difference between red and blue lines. **e** Illustration how a single minus-end directed motor and pushing microtubule tips separate microtubule asters and stabilize bipolar organization. See text for details. Source data for this figure are provided as a Source Data file.

Depletion of HSET in live cells leads to shorter spindles^10,20–22^ consistent with our findings that rather than acting only as minus directed motors, they are also part of the machinery that generates pushing forces. To dissect the contribution of the EB/HSET interaction to the force balance in mitotic spindles in live cells, we used and optogenetic H1299 cell line (π-EB1 H1299) in which all EBs could be inactivated by light (Supplementary Movie 7)^27,48^. In addition, we co-transfected the π-EB1 H1299 cells with a pair of EB and HSET molecules, where the C-terminal +TIP binding domain of EB1 was replaced with FRB (EB1ΔC-mApple-FRB), and the N-terminus tail region containing cargo and EB binding domain of HSET was truncated and replaced with FKBP (FKBP-mGFP-HSETΔN) (Fig. 5a). Without rapamycin these molecules did not contribute to the spindle size and organization as both lacked their protein interaction domains, and we confirmed that overexpression of both constructs did not affect the distribution of the metaphase spindle sizes (Supplementary Fig. 5a).

Inactivating all EBs in co-transfected metaphase cells resulted in mitotic spindles shortened by ~23% after exposure to blue light (Fig. 5b, c, Supplementary Movie 8). This was consistent with the previous result in which spindle shortening after EB inactivation was attributed to the disengagement of the cortical pulling forces in metaphase^27^. This also indicated that EB/HSET interaction may have provided pushing forces in the spindle, and when these forces were removed, the new balance of forces resulted in a shorter spindle length. To test this further, we treated cells with rapamycin before exposing them to the blue light. In this experiment, the spindle length increased ~20% comparing to non-rapamycin treated cells under blue-light stimulation (Fig. 5b, c, Supplementary Movie 9). Rapamycin treatment also made the spindle size insensitive to the blue light exposure and consistent with the spindle length in wild type cells. Consistent with our in vitro observations (Supplementary Fig. 2f), additionally engaging microtubules by the EB/HSET FRB/FKBP system did not change the rate of microtubule growth, which remained the same with and without rapamycin (Supplementary Fig. 5b).

Since pushing force generated by EB/HSET is expected to act in the antiparallel microtubule overlaps located in the spindle midzone, instead of deactivating EBs in the whole cell, we illuminated only central area. In the absence of rapamycin spindle size also decreased consistent with earlier report^27^, and decrease was restored by addition of rapamycin (Supplementary Fig. 5b, c) indicating that engaging EB/HSET system restores pushing forces deactivated in the antiparallel overlaps in the spindle midzone.

## Discussion

In this work, we developed approaches to measure mechanical contributions of individual molecules to the force balance in bipolar microtubule arrays and showed that a simple system consisting of a single minus-end directed motor and tip tracker that act synergetically with growing dynamic microtubules performs both, separation of the two microtubule asters and stabilization of their bipolar organization. We showed that this microtubule tips driven mechanism plays a crucial role in regulating spindle size in metaphase cells.

Compared to other forces generated by motors and non-motor proteins in microtubule overlaps, the microtubule tip-generated force exhibits two major differences, making this force-generation mechanism unique. The total force exerted by motors and crosslinkers that bind microtubule lattices depends on the size of the microtubule overlap where they act; larger overlaps generate more force. However, forces generated by the microtubule/EB/HSET system do not depend on the size of the overlap but on the density of the microtubule tips and the probability of the intersection between the tip and lattices of antiparallel microtubules. To understand whether the force generated at the tip/lattice intersections scales qualitatively different with the spindle size compared to the force generated in lattice/lattice overlaps, we simulated how the number of antiparallel microtubule tip/lattice interactions decreases as poles separate and compared it to the change in the overall area of the antiparallel microtubule lattice/lattice overlaps (Supplementary Fig. 6a). Our simulations show that number of tip/lattice intersections monotonically decreases as poles separate, while lattice/lattice overlaps have an optimum when the size of the spindle is approximately equal to the average length of the microtubules (Fig. 5d). These simulations show how combination of forces produced by both mechanisms in the antiparallel tip/lattice intersections and lattice/lattice overlaps provide stable bipolar steady state of the spindle (Fig. 5d, bottom). This explains how EB/HSET/microtubule system alone can build and stabilize bipolar spindle: when two asters are in proximity and the size of the spindle is small, the high density of the microtubule tips generate pushing forces that dominate over forces generated in the microtubule overlaps. As poles separate, density of the intersection between microtubule tips and lattices drops, which decreases the pushing force and leads to a balanced bipolar state (Fig. 5e).

Our study also revealed the mechanism that EB3 uses to harness the force generated by microtubule growth. The data showed that just two EB3 dimers connected by a flexible scaffold become an efficient coupling device that can be driven by the growing microtubule tip with remarkable processivity. We also discovered that when connected by a flexible scaffold, force generated by the scaffold increases exponentially with the number of EB3 dimers in the scaffold. Interpolation to the higher number of EBs showed that possibly less than ten EB3 dimers might be sufficient to harness 5 pN of force, which is approximately the maximum pushing force generated by EB/HSET system that we measured per single antiparallel microtubule pair.

The maximum force harnessed by EB/HSET from the growing microtubule tip is similar to the maximum force that a growing microtubule can generate by pushing against a barrier^30^. This was not expected given the two molecular mechanisms used for the force generation are fundamentally different. However, the upper limit of the force generation in both cases is set by the same thermodynamic constraint. The maximum force that microtubule system can generate is the energy input divided by the distance over which this energy is expended. In both cases, chemical energy is provided during attachments of the new tubulins to the microtubule tip. The distance is also determined by the microtubule growth and therefore it is also the same, suggesting that the expected maximum generated force should be equal in both mechanisms. Importantly, the upper limit of five piconewtons suggests that increasing the number of EB3 dimers in the scaffolds beyond eight to ten would not increase the force further. Therefore, given there can be many dozens of EB3 dimers at the microtubule tips during growth, we expect that only a small fraction of them would be involved in productive force generation in complexes with HSET.

In our experiments we used DNA as an artificial scaffold to investigate the effect of mechanical coupling between EBs on their ability to harness force generated by microtubule polymerization. Although DNA scaffold is very different in nature from scaffolding made by HSET, it presumably allows for the similar mechanical flexibility of the EB dimers and enables their efficient force transduction: in the DNA scaffold, neighboring EBs are separated by 23 bases of the single-stranded DNA and 25 bases of the double-stranded DNA, which results in the total distance of up to ~ 23 nm^49^. This is in good agreement with the length of the coiled neck in HSET ( ~ 25 nm) and the distance between neighbor HSETs defined by the microtubule lattice (Supplementary Fig. 6b). Therefore, both types of connection should allow for similar EB3 flexibility. Thus, although we could not control or determine the exact number of EBs in the experiments with HSET, we expect that they harness forces generated by microtubule polymerization using the same mechanism as when placed on artificial DNA scaffolds given the similarity of the overall geometry of the connection.

We also asked whether the mechanism we discovered is required for the correct assembly of the mitotic spindles in human cells. We observed that mitotic spindles shorten ~20% following rapid inactivation of EBs in metaphase and this shortening is rescued by reinstating the interaction between EB and HSET. Previously, disruption of the force balance and spindle shortening after EB inactivation was attributed to the EBs being required to engage cortical pulling forces in metaphase^27^. However, this decrease in pulling on astral microtubules can work together with the decrease in pushing on the antiparallel spindle microtubules. Interestingly, the length of the spindle decrease following EB1 inactivation is rescued completely after adding EB/HSET system, suggesting that EBs may perform their major function in mitotic spindles in the complex with HSET as was suggested in yeast^23^. These results are complementary to the previous study^27^ and show that HSET/EB can directly shift force balance by harnessing forces generated by microtubules in addition to changing microtubule dynamics^11^.

The finding that spindles shorten only 20% in metaphase suggests that after EB deactivation the system finds its new equilibrium with other mechanisms that prevent further shortening. We speculate that this equilibrium could result from the action of other motors and non-motor crosslinkers that stabilize antiparallel overlaps at this stage. Pushing forces generated by microtubule/EB/HSET system likely play even more important role at the earlier stages of the spindle formation before metaphase when pushing forces are required for the spindle poles’ separation, and the emerging antiparallel microtubule overlaps are not yet well stabilized by other molecules. This is supported by our simulations, which show that EB/HSET system can lead to the separation of two microtubule asters and stabilization of the bipolar configuration, which in live cells is likely then stabilized further by action of additional mechanisms.

Finally, our findings suggest a possible explanation of how minor changes in microtubule dynamics can lead to variations in spindle size and increased chromosome instability. We have shown that microtubule generated force directly contributes to the assembly of the mitotic spindle through a single molecular motor and a tip tracker. This system alone can result in bipolar organization of microtubule asters, but its efficiency and the resulting spindle size depend on the difference between the microtubule growth rate and the speed of the minus-end directed motor movement. Thus, changes in the microtubule dynamics and especially microtubule growth rate could affect the fidelity and robustness of the spindle assembly through the force generation at the tips of growing interpolar microtubules.

## Methods

### Cloning, expression and purification of SNAP-LZ-EB3 heterodimer

The DNA sequence of parallel Leucine zipper pair GCN4_v4 (LZ, IASRMKQLEDKVEELLSKNYHLENEVARLKKLVGECEGL) and GCN4_v4 with SNAP tag (LZ-SNAP) were customized by GeneArt (Invitrogen) into pMA plasmids. DNA sequence of EB3 were obtained from pETMz mGFP-EB3 (gift from Thomas Surrey lab) were cloned by infusion (Takara Bio) into the GCN4_v4 plasmids using the primer (EB3_LZ _for: 5’-GTATTTTCAGGGCGCCATGGCCGTCAATGTGTACTCCACATCTG-3’, EB3_LZ_rev: 5’-CACCGCCACCGGCGCCGTACTCGTCCTGGTCTTCTTGTTG-3’). The assembled EB3-LZ constructs were then subsequently cloned into a pETDuet-1 plasmid using restriction digestion and ligation. The EB3-LZ-SNAP were inserted into the first multiple cloning site containing N-terminus His-tag using the restriction enzymes (BamHI and SalI). The EB3-LZ were inserted into the second multiple cloning site containing Strep-tag using the restriction enzymes (NcoI and XhoI). The expected molecular weight for the EB3 heterodimers is ~93 kDa (EB3-LZ-SNAP: 56.24 kDa and EB3-LZ: 37.10 kDa).

The plasmid for co-expression was transformed into BL21 (DE3) pLyS cells. Single colonies were grown overnight for 12 h and picked from Ampicillin resistant LB plates. Bacteria cells were inoculated into 50 ml culture and later transferred to 4 L culture at 37 °C in 100 μg/ml Ampicillin LB media until the O.D.600 reached 0.6. The *E. coli* culture were then induced with 0.8 mM IPTG for 12 h overnight at 18 °C. The induced culture was then harvested by centrifuging at 4000 g for 30 min in a Beckman Coulter Avanti J-25 ultracentrifuge using a JLA-8.1 rotor (Beckman Coulter). The pelleted cells were resuspended in 150 ml of Lysis buffer (50 mM sodium phosphate buffer, pH 7.5, 400 mM KCl, 5 mM MgCl_2_, 0.5 mM β-mercaptoethanol) supplemented with Complete EDTA free protease inhibitor (Sigma). The cells were lysed using an microfluidizer (Microfluidics M-110L) and the lysate were cleared by centrifugation at 4 °C, 230,000 g for 30 min, in a Beckman Coulter Optima L-100XP ultracentrifuge using a Ti45 rotor (Beckman Coulter). The cleared lysate was loaded onto a HiTrap 1 ml Chelating column (Cytiva) charged with Nickel chloride using an ÄKTA pure™ 25 (Cytiva) protein purification system. The column was washed with Nickel buffer A (50 mM sodium phosphate buffer, pH 7.5, 400 mM KCl, 5 mM MgCl_2_, 5 mM Imidazole, 0.5 mM β-mercaptoethanol) for 2 column volumes and eluted with Nickel buffer B (50 mM sodium phosphate buffer, pH 7.5, 400 mM KCl, 5 mM MgCl_2_, 500 mM Imidazole, 0.5 mM β-mercaptoethanol) for 20 column volumes. Peak fractions were collected and pooled together, then loaded onto a StrepTrap 5 ml column (Cytiva), column was washed for 2 column volume with Strep Buffer A (50 mM sodium phosphate buffer, pH 7.5, 300 mM KCl, 5 mM MgCl_2_, 0.5 mM β-mercaptoethanol) and eluted with a gradient of Strep buffer B (50 mM sodium phosphate buffer, pH 7.5, 300 mM KCl, 5 mM MgCl_2_, 2.5 mM desthiobiotin, 0.5 mM β-mercaptoethanol) for 20 column volumes. Peak fractions were collected and pooled together, buffer exchanged into the Lysis buffer without protease inhibitor using a disposable PD-10 desalt column (Cytiva), added TEV protease and incubated for 12 h overnight at 4 °C. After TEV protease cleavage of the His- and Strep-tag, the sample were passed through the Nickel column and Strep column again to remove the TEV protease and protein tags. The final protein samples were pooled and concentrated to 500 μl using Vivaspin® 20 Ultrafiltration Unit with a 30 kDa molecular weight cut-off. The sample was passed through size exclusion Superdex 200 column (Cytiva) in size exclusion buffer (50 mM sodium phosphate buffer, pH 7.5, 300 mM KCl, 5 mM MgCl_2_, 0.5 mM β-mercaptoethanol), and the peak fractions were collected and pooled, the final concentration of 1 mg/ml SNAP-LZ-EB3 were aliquoted and flash frozen in Liquid Nitrogen and stored in -80 °C.

### Synthesis of DNA scaffold backbone for EB3 ensembles

DNA strands were purchased from Merck. DNA backbone was generated by splint ligation of the two sequences:

bb_fragement_1: 5′-AAGACACCTGAGGACTGTACCTATTTTGCGGCGAGAGGGACGACAGAAGGCTAATGTGGTGC-3′, and bb_fragment_2: 5′-[Phosphate]AGCATGATACGCGCAGGGGTCAATCGAAATGAGCGGAACCGGGGAATTGTAACTACTCCTAGACCAACGGATGCTTGTTTACGTGCCCATATCTATAAGCTGGATCACTTCAGTTCGGCCAAGAA-3′, using the splint_bb1_bb2: GTATCATGCTGCACCACATT. Splint ligation reaction was done by mixing DNA in equal molar ratios of 1 mM in a 20 μl reaction and annealed in 1× ligation buffer (NEB) over 4 h using a thermocycler (Bio-Rad). After ligation, reactions were diluted 100 times with PCR grade water (Thermo) and 45 μl of the diluted product was supplemented with 5 μl of 10× ligation buffer and 1 μl of T4 DNA ligase and incubated at 16 °C overnight. The ligated 187 base product was separated on a 15% TBE-urea denaturing gel and the ligated DNA band was cut out and electro-eluted from the gel piece. For the optical trapping experiments, we used modified sequence, which contained biotin: bb_fragment_2_biotin: 5′-[Phosphate]AGCATGATACGCGCAGGGGTCAATCGAAATGAGCGGAACCGGGGAATTGTAACTACTCCTAGACCAACGGATGCTTGTTTACGTGCCCATATCTATAAGCTGGATCACTTCAGTTCGGCCAAGAA[Biotin TEG] -3′. This was used for generating DNA backbone instead of bb_fragment_2.

Amino-labeled and biotin-labeled 25 base DNA complementary strands (Cy3R1: 5’-[Cy3] CCAGCTTATAGATATGGGCACGTAA[AmC6dT]T-3’, R2: 5’-TAGTTACAATTCCCCGGTTCCGCTC [AmC6dT]T-3’, R3: 5’-TCATGCTGCACCACATTAGCCTTC[AmC6dT]T-3’, R4: 5’- TAGGTACAGTCCTCAGGTGTCTT[AmC6dT]T) were coupled with SNAP-substrate NHS ester (BG-GLA-NHS, NEB). The DNA strands at 1 mM concentration were incubated with 10 mM of BG-GLA-NHS and reactions were cleaned up using a Bio-Spin P-30 column (Bio-Rad).

Depending on the required number of the EB heterodimers in the scaffold only specific strands were coupled to BG-GLA-NHS. For example, to assemble scaffolds with two EB heterodimers, R3 and R4 were coupled to BG-GLA-NHS and Cy3R1 and R2 were left unmodified.

The 187 base DNA backbone was then mixed with all four R1-R4 primers (some of which were modified to carry Snap ligand depending on the desired scaffold) at equal molar ratio and annealed in anneal buffer (10 mM Hepes, pH7.5, 50 mM NaCl, 1 mM EDTA) for 4 h using a Thermocycler (Bio-Rad). The annealed products were separated on an 8% Native TBE gel and the target bands were cut out and purified by electro-elution. The assembled scaffold was concentrated using ethanol precipitation to 1 μM. To couple EB heterodimers, the scaffold was incubated with 6 μM SNAP-LZ-EB3/LZ-EB3 heterodimers at 37 °C for 30 min in reaction buffer (20 mM KoAC pH 5.2, 100 mM NaCl, 1 mM DTT) then quickly moved on ice. The reactions were then aliquoted and flash frozen, and stored in -80 °C.

To verify EB3 heterodimer coupling to the scaffold we performed SDS-PAGE. However, we did not heat the sample in order to preserve the structure of the DNA scaffold. Presence of SDS was sufficient to break EB3 heterodimers into monomers, but it doesn’t denature dsDNA. Boiling the sample would otherwise detach heterodimers from the scaffold. Smaller weight bends are seen on the gel correspond to scaffolds with less EBs. However, the major band always corresponded to scaffolds with the expected number of attached EB heterodimers. The presence of a small fraction of scaffolds with a smaller number of EBs than expected did not affect the interpretation of the experiments.

### Considerations about DNA scaffold length and flexibility

The geometry and flexibility of the scaffold are most likely the key factors that enable EBs to harness close to 100% of the force generated by a microtubule tip. The flexibility presumably allows EB dimers that dissociate from the microtubule to diffuse locally, reach, and bind to new positions closer to the microtubule tip, while others keep the scaffold tethered to the microtubule surface (Supplementary Fig. 6c). The reason that the maximum force is reached in the presence of less than ten EB dimers is likely related to the geometry of the scaffold and the structural requirement for the transition between GDP lattice and the lattice recognized by EBs. The scaffolds only move directionally in the limited area where one lattice configuration transitions into another. EBs that dissociate in this area will have preference to bind close to the tip and therefore move directionally, while EBs positioned away from this boundary would have no preferred direction and therefore would not contribute to the force generation. The size of this transition area is likely less than 150 nm^50^, which is consistent with our estimate that increasing the size of the scaffold above ~10 EB dimers will likely push them beyond this area, thus significantly decreasing the ability of the scaffold to harness microtubule generated force.

### Expression and purification of mGFP-EB3, mCherry-HSET, mGFP-CAMSAP3

Expression and purification of mGFP-EB3 homodimers was done using the same protocol as for SNAP-LZ-EB3 heterodimer, while omitting the Strep column purification step. Expression of mCherry-HSET was done using the Bac-to-Bac insect cell expression system according to the manufacturer’s protocol (Thermo). Cell pellets from 500 ml of cell culture expressing Strep-tagged mCherry-HSET were resuspended in 40 ml of Lysis buffer (50 mM sodium phosphate pH 7.5, 300 mM KCl, 5 mM MgCl_2_, 0.5 mM ATP, 1 mM EGTA and 5 mM β-Mercaptoethanol). Cells were homogenized by douncing 40 strokes on ice using a dounce homogenizer and the lysate were spun for 45 minutes at 256,000 g, 4 °C in a Beckman Coulter Optima L-100XP ultracentrifuge using a Ti70 rotor. The cleared lysate was loaded onto a StrepTrap 5 ml column (Cytiva) and washed for 2 column volumes of Lysis buffer (50 mM sodium phosphate pH 7.5, 300 mM KCl, 5 mM MgCl_2_, 0.5 mM ATP, 1 mM EGTA and 5 mM β-mercaptoethanol), then eluted with a gradient of Elution buffer (50 mM sodium phosphate pH 7.5, 300 mM KCl, 5 mM MgCl_2_, 0.5 mM ATP, 1 mM EGTA, 2.5 mM desthiobiotin and 5 mM β-Mercaptoethanol) for 10 column volumes. Peak fractions were pooled together and incubated with TEV protease overnight at 4 °C. The sample was passed through the StrepTrap column again and flow through was collected and concentrated down to 500 μl before the size exclusion chromatography. Sample were injected into size exclusion column Superose 6 10/300 (Cytiva) equilibrated with Size exclusion buffer (50 mM sodium phosphate, pH7.5, 300 mM KCl, 1 mM MgCl_2_, 0.1 mM ATP, 1 mM EGTA and 5 mM β-mercaptoethanol) peak fractions were pooled and concentrated to 500 μl before flash frozen and stored in -80 °C.

Strep-tagged mGFP-CAMSAP-3 were expressed in HEK293T cell lines, mammalian expression plasmid for mGFP-CAMSAP-3 was a gift from Dr. Anna Akmanova’s lab. HEK293T cells were seeded in Dulbecco’s Modified Eagle’s Medium (Gibco) supplemented with 10% FBS (Gibco) and Pen/Strep (Gibco), at 20 % confluency in four 15 cm tissue culture dishes (Corning). Cells were transfected using lipofectamine 3000 reagent (Thermo) according to the manufacturer’s protocol. Growth media was exchanged after 24 h and cells were checked for protein expression using a fluorescent cell imager (Bio-Rad). Cells were harvested after another 24 h in cold PBS before purification. Purification were done by resuspending the cells in 2 ml of lysis buffer (50 mM HEPES, pH 7.4, 300 mM NaCl, 0.5% Triton X-100, 1 mM MgCl_2_, 0.1 mM ATP and 1 mM EGTA) supplemented with Complete EDTA free protease inhibitor (Sigma) and incubated on ice for 15 min. Lysate were then spun at 25,300 g for 20 min at 4 °C. Supernatant were collected and mixed with Strep-Tactin sepharose (IBA-Lifesciences) washed with lysis buffer and eluted with elution buffer (50 mM HEPES, pH 7.4, 150 mM NaCl, 1 mM MgCl_2_, 1 mM EGTA, 0.05% Triton X-100, 2.5 mM desthiobiotin, 0.1 mM ATP, and 2.5 mM DTT). The eluted samples were aliquoted and flash frozen and stored in -80 °C.

### Generating DIG-labeled GMPCPP stabilized microtubule seeds

Digoxigenin labeled GMPCPP microtubule seeds were made by mixing 12 μM of 15% Digoxigenin labeled tubulin, and 2 μM of 20% Alexa 647 labeled tubulin in 1 mM GMPCPP, 1 mM DTT and BRB80. Reactions were incubated at 37 °C for 1 h before spun down at 278,000 g using a TLA-100 rotor. The Pellet was rinsed twice using warm BRB80 plus DTT and the microtubule seeds were checked using a fluorescent TIRF microscope. The GMPCPP microtubule seeds can be stored at room temperature protected from light for 2 weeks. Labeled tubulin was made by chemical crosslinking of Digoxigenin-NHS ester (Enzo, ENZ-45022) or Alexa647-NHS-ester (Thermo) to microtubules and cycling tubulin using standard protocols, and the final labeling ratio was determined using NanoDrop (Thermo). Tubulin was purified from porcine brains. Tubulin purification and modification was done using standard procedures^51,52^.

### Dual-trap optical tweezers experiment

Two bead experiments were performed on LUMICKS C-Trap system, which has a microfluidic flow-cell (LUMICKS) containing five laminar flow channels. The system was equipped with a three-color confocal imaging and a dual optical trap. We used four of the five channels in the C-Trap to perform our experiment. Before the experiment, the flow cell surface was washed with PBS and passivated with 5% Pluronic F-127 for 10 minutes before washing again with Room-temperature BRB80 buffer. Anti-digoxigenin coated polystyrene beads (2 μm diameter, Bangs Lab) were flown into channel 1 and GMPCPP stabilized microtubule seeds labeled with digoxigenin and Alexa647 were flow into channel 2. Channel 3 contained only BRB80 buffer and channel 4 was filled with reaction buffer supplemented with 14 μM unlabeled tubulin, 2 μM of 20% Alexa 647 labeled tubulin, 10 nM CAMSAP3, 100 nM EB3 and 20 nM HSET in 80 mM PIPES, 75 mM KoAC, 15 mM KCl, 1 mM MgCl_2_, 1 mM EGTA, 1 mM DTT, 1 mM ATP and 1 mM GTP. Addition of CAMSAP3 blocked the microtubule growth at the minus ends and ensured that all microtubule dynamics occurred only at their plus ends. During the experiment, the beads were trapped in channel 1 with the dual-optical tweezers at a constant flow generated by ~0.03 bar pressure, then moved immediately to channel 2 and left to incubate for a few seconds to bind the seeds. Time and concentration of GMPCPP seeds were chosen such that few seeds were attached for the beads as shown in Supplementary Movie 1 and 3, which was done to minimize the number of microtubule intersections. Higher concentration of seeds resulted in many more microtubules nucleated at the beads, but were generally not used here (Supplementary Movie 10). The beads were then moved to channel 3 to remove unbound seeds and the seeds coverage was visualized by fluorescent confocal scanning. The beads with microtubule seeds bound were then moved slowly to channel 4. The flow stretched the microtubules growing from the seeds, which allowed to estimate their length. Next, the flow was switched off and the beads were placed at a distance at which microtubules growing form the opposite beads were expected to engage (typically 6 to 12 microns). Force recording was started simultaneously with imaging in three fluorescent channels. Forces were recorded for 10 minutes before flow was switched back on and beads were discarded to start a new round of experiment.

Images were processed Fiji (ImageJ v 2.14) and data was analyzed in OriginPro 2021. Direction of force was determined by the increase or decrease in the distance between beads. The rate of microtubule sliding was determined by a linear curve fit of the slope of each force peak.

### Coverslips preparation for the in vitro experiments

24 × 60 mm glass coverslips (VWR) were cleaned by sonication for 15 minutes each sequentially in Milli-Q water, 100% Acetone, 100% ethanol, 1 M KOH. After cleaning, the coverslips were dried with filtered Nitrogen gas and the glass surface were activated by a plasma cleaner (Corning) for 3 minutes in air at maximum power (3 W). The coverslips were then transferred to a clean glass container and submerged in a mixture of 1:20 Dichloromethylsilane:Heptane, then incubated at room temperature for 1 h. After incubation, the solution was decanted and the hydrophobic coverslips were sonicated for 5 minutes subsequently in chloroform, Milli-Q water, then chloroform again. The Hydrophobic coverslips are air-dried and stored in room temperature.

### Microtubule buckling experiments

Experiments were done using a flow cell assembled by separating silanized hydrophobic coverslips and cleaned glass slides (VWR) by double sided tape (3 M). The channels were first incubated with anti-digoxigenin nanobodies (AntiDig-Fab, Roche) followed by passivation using 5% Pluronic F-127 for 15 min and blocked further with 1% BSA in BRB80. 1: 50 dilution of Digoxigenin labeled GMPCPP seeds were pipetted into one end of the flow cell channel and blotted with filter paper on the other end of the channel, the seeds were left incubating in the channel for 1 min before washing two times with 100 μL warm BRB80 buffer. 50 μl of pre-warmed reaction mixture containing 14 μM of unlabeled tubulin, 2 μM of 20% Alexa 647 labeled tubulin, 100 nM EB3 and various concentrations of HSET in 80 mM PIPES, 75 mM KoAC, 15 mM KCl, 1 mM MgCl_2_, 1 mM EGTA, 1 mM DTT, 1 mM ATP and 1 mM GTP were flown into the channel immediately before imagining. The flow cells were then mounted onto the microscope stage where the objective was warmed to 37 °C with an objective heater (TOKAI HIT). Images were recorded for 300 seconds at a frame rate of 1 Hz on. Fluorescence was split using OptoSplit (Cairn Research) on two Sona sCMOS cameras (Andor). Three color channels were recorded simultaneously on two cameras by synchronizing laser shutters with camera’s fire tiggers to produce alternating images of one or the other color on one of the cameras. Without CAMSAP3, antiparallel microtubule bundles were identified by the direction of HSET movement towards the minus end of each microtubule. Images were later processed and analyzed using Fiji (ImageJ v2.14) and MATLAB 2019.

### Fluorescence recovery after photobleaching (FRAP)

FRAP experiment was done by imaging microtubule dynamics as described above for the microtubule buckling experiment. Under normal imaging conditions, we typically used 488 nm laser (50 mW max power) and 561 nm laser (150 mW) set to 20%. For the simultaneous photobleaching of both EB3 and HSET both 488 and 561 lasers were set to 100% for 50 frames. Recovery was monitored by changing laser powers back to 20% and imaging further. Images were later processed and analyzed using Fiji (ImageJ v2.14) and MATLAB 2019.

### Analysis of the microtubule shapes and forces

Microtubules were digitized using Fiji (ImageJ v2.14) multipoint function. Minimum seven points were used per single microtubule, and individual frames were analyzed independently with 5 frame (5 s) interval between them. Coordinates of the points were then transferred to MATLAB. The shape of the microtubule was interpolated using smoothing spline and the curvature as a function of the position along the microtubule was calculated using:

1 $$\kappa=\frac{\left|y{\prime} {\prime} \right|}{{\left(1+{y{\prime} }^{2}\right)}^{3/2}}$$

Where $y(x)$ describes the spline interpolated shape of the microtubule.

Microtubule buckling force was calculated as:

2 $$F=12\frac{{k}_{B}T\,{L}_{P}}{{L}^{2}}$$

Where $L$ is the microtubule contour length and ${L}_{P}$ is the persistence length. In all our experiments, we used ${L}_{P}$ = 4.8 mm^53^. We determined buckling force 15 – 20 seconds after the initial engagement, which corresponded to the first time that microtubule underwent bending. Thus, we assumed, microtubules did not accumulate any lattice defects that could decrease their persistence length, which we assumed independent of the microtubule length. Microtubule length $L$ was determined as the length of the microtubule from the tip to the seed at the moment when microtubule became visibly bent. Images of microtubules were digitized and their curvature calculated by custom software written in MATLAB 2019.

### Single-Molecule Fluorescence TIRF microscopy of EB-DNA scaffold movement

Flow cells used in this experiment were assembled and passivated as described in the previous section on microtubule buckling experiment. 50 μl of pre-warmed reaction mixture containing 14 μM of unlabeled tubulin, 2 μM of 20% Alexa 647 labeled tubulin, 1 nM of DNA scaffold EB3 mixture in 80 mM PIPES, 75 mM KoAC, 15 mM KCl, 1 mM MgCl_2_, 1 mM EGTA, 1 mM DTT and 1 mM GTP were flow into the channel immediately before imagining. The flow cells were then mounted onto the microscope stage where the objective was warmed to 37 °C. Images were recorded for 300 seconds, at a frame rate of 1 Hz on two Sona sCMOS cameras (Andor) for dual color imagining, with an exposure time of 100 ms. Images were later processed and analyzed by Image J.

### DNA scaffold force measurement

Since beads were used in these experiments, the flow had to be more tightly controlled. To achieve this, we used flow cells assembled using parafilm sandwiched between a silanized cover slip and a glass slide. The glass slide had metal tubing (New England Small Tube Corp) glued to it on each end using epoxy glue (POXIPOL). Metal connectors were connected by tubing to the syringe pump (Harvard Apparatus). ~1 nM of biotinylated DNA scaffold coupled to EB3 ensembles were incubated with 1:100 dilution of (1 μm diameter, Bangs Lab) Streptavidin polystyrene beads for 15 min on ice in PBS, pH 7.4 and 2% BSA at a total reaction volume of 50 μl. Beads were then washed twice with ice cold PBS and resuspended in 50 μL PBS. 1 μl of EB3 DNA scaffold ensemble coupled beads were mixed with 50 μl of room-temperature reaction mixture containing 14 μM of unlabeled tubulin, 2 μM of 20% Alexa 647 labeled tubulin, in 80 mM PIPES, 75 mM KoAC, 15 mM KCl, 1 mM MgCl_2_, 1 mM EGTA, 1 mM DTT and 1 mM GTP before flowing into the flow cell.

Force measurement was performed by trapping a bead using a single-optical trap (JPK, Nanotracker) and holding it ~100 nm above the coverslip surface. The bead was moved and placed in front of a growing microtubule and the force were recorded at 2 kHz with a typical trap stiffness of 0.02 pN/nm.

To ensure that movement of the bead was due to a single scaffold only, we diluted the scaffolds such that only ~20% of beads interacted with growing microtubule tips (*n* = 42 out of 200). Assuming binding of the scaffolds to beads is a random process governed by Poisson distribution and considering that ~80% of beads did not have a single active scaffold, this suggests that ~ 18% of all beads in our experiments must have had a single active scaffold and only ~2% have had two of more scaffolds. Thus, out of all signals detected ~90% must have been generated by the movement of the single scaffold.

Fluorescent imaging at 647 nm wavelength was performed simultaneously with the force recording using a Andor iXon EMCCD camera at 1 Hz, 100 ms exposure time.

Force curves were later processed using MATLAB 2019 or OriginPro 2021 software. The force curves were first smoothed down to 20 Hz then fitted with sigmoidal logistic function:

3 $$y={A}_{1}+\frac{{A}_{2}-{A}_{1}}{1+{\left(\frac{x}{{x}_{0}}\right)}^{p}}$$

The force was determined as $F={A}_{1}-{A}_{2}$. Only signals in which increase in the force signal agreed with the microtubule growth rate determined from the corresponding fluorescent imaging recording were selected for the further processing.

### Cytosim simulations

The modeling software Cytosim^54^ was used to model the effect of HSET and EB/HSET system on the organization of two microtubule asters into bipolar structure. Cytosim models microtubules as fibers with rigidity 20 pN·µm/rad and diffusing cross-linking units as particles undergoing Brownian motion in a viscous medium described by over-damped Langevin equations. Viscosity was set in all simulations to 0.01 pN·s/µm^2^. Simulations were run with a time-step of 5 ms for 1000 s. The simulation space was a circle with diameter 60 µm. To mimic the spindle, ~100 growing microtubules (0.75 µm in length) were initialized from two asters (diameter 1 µm) separated by 2 µm. Microtubule dynamic instability was simulated as a two-state model defined by a catastrophe frequency (k_cat_ = 0.005 s^−1^ or 0.06 s^−1^ for slower and faster growing microtubules respectively), a rescue frequency (k_rescue_ = 0.06 s^−1^ in all simulations), a constant shrinkage speed (v_s_), and a force-dependent growth speed (v_g_). The growth rate was assumed to be reduced by an antagonistic force (f_a_) by an exponential factor exp (f_a_ / f_g_), where f_g_ is a characteristic growth force which was set to be 1.67 pN. Once a shrinking microtubule’s length is reduced below 100 nm, a new growing microtubule is initialized on that aster.

HSET and EB/HSET units were modeled as two hands linked by a Hookean spring. Hands can bind separate microtubules, cross-link the microtubules and apply forces to the microtubules. For the EB/HSET motors, the unit representing HSET bound the microtubule lattice and moved towards the minus end. The unit representing the EB bound and tracked the microtubule tip and could resist external forces up to 5 pN as suggested by our experiments. Thus, in this simplified model, the EB domain of the EB/HSET system represented the collective action of multiple EBs that remained bound at the microtubule tip as a scaffold stabilized by their interaction with the other microtubule via HSET. Thus, EB/HSET complex effectively combined the minus-end and plus-end directed activities in one system (Fig. 4b, c). Such hypothetical motors were theoretically predicted to be able to generate stable bipolar configurations earlier^55^ but experimental evidence for their existence has been lacking. Our experimental data present evidence that they may indeed exist and be formed by combined action of separate molecules: while HSET moves to the minus-end, EBs on the tips of the growing microtubule end move towards the plus-end direction. Thus, the minus-end directed action of HSET drives tips of microtubules towards the opposite pole, but only for growing microtubules.

The association rate was assumed to be k_on_ = 10 s^−1^ for all motors. The dissociation rate was force dependent as: k  =  k_off_ exp (f_load_ / f_stall_). We used k_off_ = 0.1 s^−1^ and assumed f_stall_ either 5 pN or 0.1 pN as discussed in the text. HSET was modeled as a processive motor directed towards the minus end of microtubules. The motor was characterized by its unloaded speed (v_m_) and a stall force (f_stall_). Load forces (f_load_) reduce the motor speed; v =  v_m_ (1 + f_load_ ⋅ d / f_stall_). EB was modeled as a plus-end tracking unit, binding specifically to a region near the plus-end of microtubules and tracking the plus-end. In all simulations, we assumed that the total number of motors and tip trackers was constant and varied their ratio in the volume containing two microtubule asters. Movie frames were generated using the *play* function of Cytosim and assembled into an AVI file in ImageJ. Aster positions were extracted using the *report* function of Cytosim and analyzed using a Jupyter notebook. Inter-aster distances were plotted against time.

### Analysis of microtubule buckling using Cytosim simulations

We also used Cytosim to investigate buckling of microtubules in our TIRF assays. Two microtubules were positioned opposite each other in a circle with 30 µm radius. Parameters of the HSET and EB/HSET units were as described in the previous section. To model passive crosslinkers that in real experiments may correspond to inactive crosslinker or dead HSET motors, we copied properties of HSET units and set their movement speed to zero. In the simulations in Supplementary Fig. 7 number of HSET units was chosen 500, number of EB/HSET units 300 and number of passive crosslinkers 350. Supplementary Fig. 7a,b are snapshots from Supplementary Movies 11 and 12 showing that as microtubule grow and engage in the presence of HSET buckling only occurs in the presence of EB/HSET units. We have analyzed curvatures of microtubule following engagement using the same algorithm as we used to analyzing curvatures of microtubules from TIRF experiments (See section “Analysis of the microtubule shapes and forces” above). Supplementary Fig. 7c shows that presence of ~ 30% inactive crosslinkers does not affect ability of EB/HSET to generate pushing force and buckle microtubules.

### Simulation of the number of antiparallel tip/lattice and lattice/lattice intersections

To simulate data on Fig. 5d, we assumed two asters of microtubules are positioned at a variable length between them. The number of microtubules per aster was set to 200. For each spindle size, we generated 1000 configurations with random microtubule lengths picked from exponential distribution with the average microtubule length equal to the pole-pole distance (further increase in microtubule length mostly increases number or parallel but not antiparallel overlaps). Angles of microtubules were assumed also random. After each configuration was generated, we counted number of antiparallel tip/lattice and lattice/lattice intersections. Number of tip/lattice intersection is plotted directly in Fig. 5d. Next, we converted number of lattice/lattice intersection into the amount of the antiparallel overlaps. Since these simulations did not have any motors that could crosslink and bend microtubules, all microtubule appear straight. We assumed that in spindles all intersection between antiparallel microtubules will be converted into overlaps and we counted the area of the overlap by assuming that for each antiparallel lattice/lattice interaction it was increased by the length of the microtubule participating in this interaction from the intersection until the end of the microtubules. Thus, middle graph in Fig. 5d shows total length of microtubules determined by this method participating in antiparallel overlaps. Lines on the Fig. 5d are trendlines that were obtained as fits with functions that capture the overall shape of the curve. Top figure is fit with double exponential curve, middle figure with rational polynomial of rank 2. Next, we assumed that the number of antiparallel tip/lattice interactions is proportional to the pushing force generated by the tips acting on the spindles. The amount of antiparallel lattice/lattice overlaps is proportional to the number of motors that can bind in these overlaps and, therefore, the total force generated in overlaps. Bottom figure in Fig. 5 show difference between the trendlines obtained for the pushing and pulling forces with equal weights. Different weights would affect exact shape of the total force and position of the steady state.

### Optogenetic inhibition of EB1/HSET interaction on π-EB1 cell lines

π-EB1 H1299 cell lines were a kind gift from Torsten Wittmann. Cells were seeded in a 35 mm high glass bottom dish (ibidi) at 80% confluency. The cells were double transfected with pLVX-EB1ΔC-mApple-FRB and pLVX-FKBP-mGFP-HSETΔN using Lipofectamine 3000 (Thermo). Cells were grown in RPMI-1640 media (Gibco) supplemented with 10% FCS (Gibco) and 1% Pen-Strep in a tissue culture incubator supplied with 5% CO2 at 37 °C for 24 h. 1 h before imaging, the media is replaced with Hanks’ Balanced Salt Solution (HBSS, Gibco) with 10% FCS. 10 µM of MG132 and 0.1% DMSO (Sigma-Aldrich) were added to the media 10 min before imaging for cell cycle arrest at metaphase. For cells treated with Rapamycin, Rapamycin was added to a final concentration of 100 nM in Hanks’ Balanced Salt Solution with 10% FCS and 10 µM of MG132, and the media is added to the cells 10 min before imaging. Imaging was performed no more than 4 h after MG132 treatment.

Imaging was carried on a Nikon CSU-W1 Spinning Disk confocal system using a 60 × 1.49 NA oil immersion lens (Nikon). Cells at metaphase expressing both EB1ΔC-mApple-FRB and FKBP-mGFP-HSETΔN were selected by equal intensity of both red and green fluorescence. For blue light illumination, time-lapsed imaging acquisition was carried out at 488 nm and 561 nm wavelength, with a 0.1 second exposure time. Blue-light illumination was triggered by camera with a 1 second delay after camera exposure and 0.4 seconds blue light intervals, z-stack were taken in the range of 15 µm and 1 µm per slice.

Images were further processed in ImageJ. The length of the spindle was determined from the pole-to-pole distance using all optical sections. The first timepoint of each time-lapsed Movie was taken before blue-light stimulation (in the dark) and the rest of the sequence was taken in the presence of the blue light.

Localized photoactivation was done using a Zeiss LSM880 confocal system with 60 × 1.49 NA oil lens. For blue light activation, mitotic cells were activated by selecting a region (using “Regions” function in ZEN Black software) at the midzone of the spindle and stimulated with 50% 488 nm laser power throughout the time-lapse recording. Movies were recorded in the mApple channel (pLVX-EB1ΔC-mApple-FRB) with a 0.1 second exposure time for 70 seconds, and were later processed with Fiji (ImageJ v2.14).

### Statistics & Reproducibility

All experiments were repeated at least three times independently on different days. Typically, number of experiments was significantly larger. Exact numbers are shown on the figures and figure legends. The sample size was not predetermined, and data was collected until statistical significance was reached. No data were excluded from analysis except in force measurements with single scaffolds. In those experiments traces were excluded if force increase was significantly slower than the average microtubule growth rate in those conditions. Investigators were not blinded. All samples were prepared on the day of the measurement by the same author collecting the data. Single-molecule data with the same conditions are inherently randomized and no other special randomization was implemented.

### Reporting summary

Further information on research design is available in the Nature Portfolio Reporting Summary linked to this article.

## Supplementary information

Supplementary Information

Description of Additional Supplementary Information

Supplementary Movie 1

Supplementary Movie 2

Supplementary Movie 3

Supplementary Movie 4

Supplementary Movie 5

Supplementary Movie 6

Supplementary Movie 7

Supplementary Movie 8

Supplementary Movie 9

Supplementary Movie 10

Supplementary Movie 11

Supplementary Movie 12

Reporting Summary

Transparent Peer Review file

## Source data

Source Data

## Data Availability

Data used for the generation of figures is provided with this paper as a Source Data file. The rest of the raw data is available from the authors upon request. Source data are provided with this paper.

## Code availability

Custom code used for the data processing and simulations is freely available in the GitHub repository (https://github.com/FrancisCrickInstitute/MT_Force_Spindle).

## References

[1] McIntosh, J. R., Molodtsov, M. I. & Ataullakhanov, F. I. Biophysics of mitosis. *Q Rev. Biophys.* **45**, 147–207 (2012). 22321376 10.1017/S0033583512000017 PMC4433171

[2] Forth, S. & Kapoor, T. M. The mechanics of microtubule networks in cell division. *J. Cell Biol.* **216**, 1525–1531 (2017). 28490474 10.1083/jcb.201612064 PMC5461028

[3] Valdez, V. A., Neahring, L., Petry, S. & Dumont, S. Mechanisms underlying spindle assembly and robustness. *Nat. Rev. Mol. Cell Biol.* **24**, 523–542 (2023). 10.1038/s41580-023-00584-0 PMC10642710 36977834

[4] Hannabuss, J. et al. Self-Organization of Minimal Anaphase Spindle Midzone Bundles. *Curr. Biol.* **29**, 2120–2130.e2127 (2019). 31231047 10.1016/j.cub.2019.05.049 PMC6616649

[5] Subramanian, R. et al. Insights into antiparallel microtubule crosslinking by PRC1, a conserved nonmotor microtubule binding protein. *Cell* **142**, 433–443 (2010). 20691902 10.1016/j.cell.2010.07.012 PMC2966277

[6] Lansky, Z. et al. Diffusible crosslinkers generate directed forces in microtubule networks. *Cell* **160**, 1159–1168 (2015). 25748652 10.1016/j.cell.2015.01.051

[7] Neahring, L., Cho, N. H. & Dumont, S. Opposing motors provide mechanical and functional robustness in the human spindle. *Dev. Cell* **56**, 3006–3018.e3005 (2021). 34614397 10.1016/j.devcel.2021.09.011 PMC8578449

[8] Kleylein-Sohn, J. et al. Acentrosomal spindle organization renders cancer cells dependent on the kinesin HSET. *J. Cell Sci.* **125**, 5391–5402 (2012). 22946058 10.1242/jcs.107474

[9] Goshima, G., Nedelec, F. & Vale, R. D. Mechanisms for focusing mitotic spindle poles by minus end-directed motor proteins. *J. Cell Biol.* **171**, 229–240 (2005). 16247025 10.1083/jcb.200505107 PMC2171195

[10] Cai, S., Weaver, L. N., Ems-McClung, S. C. & Walczak, C. E. Kinesin-14 family proteins HSET/XCTK2 control spindle length by cross-linking and sliding microtubules. *Mol. Biol. Cell* **20**, 1348–1359 (2009). 19116309 10.1091/mbc.E08-09-0971 PMC2649268

[11] Ogren, A. et al. Kinesin-14 motors participate in a force balance at microtubule plus-ends to regulate dynamic instability. *Proc. Natl. Acad. Sci. USA.* **119**, e2108046119 (2022). 10.1073/pnas.2108046119 PMC8872730 35173049

[12] Fink, G. et al. The mitotic kinesin-14 Ncd drives directional microtubule-microtubule sliding. *Nat. Cell Biol.* **11**, 717–723 (2009). 19430467 10.1038/ncb1877

[13] Braun, M., Drummond, D. R., Cross, R. A. & McAinsh, A. D. The kinesin-14 Klp2 organizes microtubules into parallel bundles by an ATP-dependent sorting mechanism. *Nat. Cell Biol.* **11**, 724–730 (2009). 19430466 10.1038/ncb1878

[14] Hentrich, C. & Surrey, T. Microtubule organization by the antagonistic mitotic motors kinesin-5 and kinesin-14. *J. Cell Biol.* **189**, 465–480 (2010). 20439998 10.1083/jcb.200910125 PMC2867311

[15] Ludecke, A., Seidel, A. M., Braun, M., Lansky, Z. & Diez, S. Diffusive tail anchorage determines velocity and force produced by kinesin-14 between crosslinked microtubules. *Nat. Commun.* **9**, 2214 (2018). 29880831 10.1038/s41467-018-04656-0 PMC5992172

[16] Braun, M. et al. Changes in microtubule overlap length regulate kinesin-14-driven microtubule sliding. *Nat. Chem. Biol.* **13**, 1245–1252 (2017). 29035362 10.1038/nchembio.2495 PMC5700410

[17] Norris, S. R. et al. Microtubule minus-end aster organization is driven by processive HSET-tubulin clusters. *Nat. Commun.* **9**, 2659 (2018). 29985404 10.1038/s41467-018-04991-2 PMC6037785

[18] Hepperla, A. J. et al. Minus-end-directed Kinesin-14 motors align antiparallel microtubules to control metaphase spindle length. *Dev. Cell* **31**, 61–72 (2014). 25313961 10.1016/j.devcel.2014.07.023 PMC4197412

[19] Henkin, G., Chew, W. X., Nedelec, F. & Surrey, T. Cross-linker design determines microtubule network organization by opposing motors. *Proc. Natl Acad. Sci. USA* **119**, e2206398119 (2022). 35960844 10.1073/pnas.2206398119 PMC9388136

[20] van Heesbeen, R. G., Tanenbaum, M. E. & Medema, R. H. Balanced activity of three mitotic motors is required for bipolar spindle assembly and chromosome segregation. *Cell Rep.* **8**, 948–956 (2014). 25127142 10.1016/j.celrep.2014.07.015

[21] Carlier-Grynkorn, F., Fachinetti, D. & Tran, P. T. Kinesin-14 HSET may not oppose kinesin-5 Eg5 activity in RPE-1 cells. *Micro. Publ. Biol.* **2022**, 17912 (2022). 10.17912/micropub.biology.000623 PMC9393730 36004005

[22] Bennabi, I. et al. Shifting meiotic to mitotic spindle assembly in oocytes disrupts chromosome alignment. *EMBO Rep.* **19**, 368–381 (2018). 29330318 10.15252/embr.201745225 PMC5797964

[23] Kornakov, N. & Westermann, S. Systematic analysis of microtubule plus-end networks defines EB-cargo complexes critical for mitosis in budding yeast. *Mol. Biol. Cell* **34**, ar37 (2023). 36884292 10.1091/mbc.E23-02-0054 PMC10162426

[24] Molodtsov, M. I. et al. A force-induced directional switch of a molecular motor enables parallel microtubule bundle formation. *Cell* **167**, 539–552.e514 (2016). 27716509 10.1016/j.cell.2016.09.029

[25] Akhmanova, A. & Steinmetz, M. O. Control of microtubule organization and dynamics: two ends in the limelight. *Nat. Rev. Mol. Cell Biol.* **16**, 711–726 (2015). 26562752 10.1038/nrm4084

[26] Goshima, G., Wollman, R., Stuurman, N., Scholey, J. M. & Vale, R. D. Length control of the metaphase spindle. *Curr. Biol.* **15**, 1979–1988 (2005). 16303556 10.1016/j.cub.2005.09.054

[27] Dema, A., van Haren, J. & Wittmann, T. Optogenetic EB1 inactivation shortens metaphase spindles by disrupting cortical force-producing interactions with astral microtubules. *Curr. Biol.* **32**, 1197–1205.e1194 (2022). 35090591 10.1016/j.cub.2022.01.017 PMC8930524

[28] Green, R. A., Wollman, R. & Kaplan, K. B. APC and EB1 function together in mitosis to regulate spindle dynamics and chromosome alignment. *Mol. Biol. Cell* **16**, 4609–4622 (2005). 16030254 10.1091/mbc.E05-03-0259 PMC1237068

[29] Draviam, V. M., Shapiro, I., Aldridge, B. & Sorger, P. K. Misorientation and reduced stretching of aligned sister kinetochores promote chromosome missegregation in EB1- or APC-depleted cells. *EMBO J.* **25**, 2814–2827 (2006). 16763565 10.1038/sj.emboj.7601168 PMC1500857

[30] Dogterom, M. & Yurke, B. Measurement of the force-velocity relation for growing microtubules. *Science* **278**, 856–860 (1997). 9346483 10.1126/science.278.5339.856

[31] Garzon-Coral, C., Fantana, H. A. & Howard, J. A force-generating machinery maintains the spindle at the cell center during mitosis. *Science* **352**, 1124–1127 (2016). 27230381 10.1126/science.aad9745 PMC6535051

[32] Jain, I., Rao, M. & Tran, P. T. Reliable and robust control of nucleus centering is contingent on nonequilibrium force patterns. *iScience* **26**, 106665 (2023). 37182105 10.1016/j.isci.2023.106665 PMC10173738

[33] Meaders, J. L., de Matos, S. N. & Burgess, D. R. A Pushing Mechanism for Microtubule Aster Positioning in a Large Cell Type. *Cell Rep.* **33**, 108213 (2020). 33027648 10.1016/j.celrep.2020.108213

[34] Zhao, T., Graham, O. S., Raposo, A. & St Johnston, D. Growing microtubules push the oocyte nucleus to polarize the Drosophila dorsal-ventral axis. *Science* **336**, 999–1003 (2012). 22499806 10.1126/science.1219147 PMC3459055

[35] Balzer, E. M. et al. Physical confinement alters tumor cell adhesion and migration phenotypes. *FASEB J.* **26**, 4045–4056 (2012). 22707566 10.1096/fj.12-211441 PMC3448771

[36] Rodriguez-Garcia, R. et al. Mechanisms of Motor-Independent Membrane Remodeling Driven by Dynamic Microtubules. *Curr. Biol.* **30**, 972–987.e912 (2020). 32032506 10.1016/j.cub.2020.01.036 PMC7090928

[37] Alkemade, C. et al. Cross-linkers at growing microtubule ends generate forces that drive actin transport. *Proc. Natl Acad. Sci. USA* **119**, e2112799119 (2022). 35271394 10.1073/pnas.2112799119 PMC8931237

[38] van Doorn, G. S., Tanase, C., Mulder, B. M. & Dogterom, M. On the stall force for growing microtubules. *Eur. Biophys. J.* **29**, 2–6 (2000). 10826773 10.1007/s002490050245

[39] Maan, R. et al. Multivalent interactions facilitate motor-dependent protein accumulation at growing microtubule plus-ends. *Nat. Cell Biol.* **25**, 68–78 (2023). 36536175 10.1038/s41556-022-01037-0 PMC9859754

[40] Volkov, V. A., Huis In ‘t Veld, P. J., Dogterom, M. & Musacchio, A. Multivalency of NDC80 in the outer kinetochore is essential to track shortening microtubules and generate forces. *Elife* **7**, e36764 (2018). 10.7554/eLife.36764 PMC5940359 29629870

[41] Akiyoshi, B. et al. Tension directly stabilizes reconstituted kinetochore-microtubule attachments. *Nature* **468**, 576–579 (2010). 21107429 10.1038/nature09594 PMC3108429

[42] Powers, A. F. et al. The Ndc80 kinetochore complex forms load-bearing attachments to dynamic microtubule tips via biased diffusion. *Cell* **136**, 865–875 (2009). 19269365 10.1016/j.cell.2008.12.045 PMC2749323

[43] Monda, J. K. et al. Microtubule tip tracking by the spindle and kinetochore protein Ska1 requires diverse tubulin-interacting surfaces. *Curr. Biol.* **27**, 3666–3675 (2017). 29153323 10.1016/j.cub.2017.10.018 PMC5726585

[44] Braun, M. et al. The human kinesin-14 HSET tracks the tips of growing microtubules in vitro. *Cytoskeleton (Hoboken)* **70**, 515–521 (2013). 24039245 10.1002/cm.21133

[45] Gittes, F., Meyhofer, E., Baek, S. & Howard, J. Directional loading of the kinesin motor molecule as it buckles a microtubule. *Biophys. J.* **70**, 418–429 (1996). 8770218 10.1016/S0006-3495(96)79585-1 PMC1224940

[46] Sironi, L. et al. Automatic quantification of microtubule dynamics enables RNAi-screening of new mitotic spindle regulators. *Cytoskeleton (Hoboken)* **68**, 266–278 (2011). 21491614 10.1002/cm.20510

[47] Lugo, C. A., Saikia, E. & Nedelec, F. A Typical workflow to simulate cytoskeletal systems. *J. Vis. Exp.* **194**, e64125 (2023). 10.3791/64125 37092845

[48] van Haren, J. et al. Local control of intracellular microtubule dynamics by EB1 photodissociation. *Nat. Cell Biol.* **20**, 252–261 (2018). 29379139 10.1038/s41556-017-0028-5 PMC5826794

[49] Ambia-Garrido, J., Vainrub, A. & Pettitt, B. M. A model for Structure and Thermodynamics of ssDNA and dsDNA Near a Surface: a Coarse Grained Approach. *Comput Phys. Commun.* **181**, 2001–2007 (2010). 20957064 10.1016/j.cpc.2010.08.029 PMC2955266

[50] Rickman, J., Duellberg, C., Cade, N. I., Griffin, L. D. & Surrey, T. Steady-state EB cap size fluctuations are determined by stochastic microtubule growth and maturation. *Proc. Natl Acad. Sci. USA* **114**, 3427–3432 (2017). 28280102 10.1073/pnas.1620274114 PMC5380103

[51] Castoldi, M. & Popov, A. V. Purification of brain tubulin through two cycles of polymerization-depolymerization in a high-molarity buffer. *Protein Expr. Purif.* **32**, 83–88 (2003). 14680943 10.1016/S1046-5928(03)00218-3

[52] Hyman, A. et al. Preparation of modified tubulins. *Methods Enzymol.* **196**, 478–485 (1991). 2034137 10.1016/0076-6879(91)96041-o

[53] Gittes, F., Mickey, B., Nettleton, J. & Howard, J. Flexural rigidity of microtubules and actin filaments measured from thermal fluctuations in shape. *J. Cell Biol.* **120**, 923–934 (1993). 8432732 10.1083/jcb.120.4.923 PMC2200075

[54] Nedelec, F. & Foethke, D. Collective Langevin dynamics of flexible cytoskeletal fibers. *N. J. Phys.* **9**, 427 (2007).

[55] Nedelec, F. Computer simulations reveal motor properties generating stable antiparallel microtubule interactions. *J. Cell Biol.* **158**, 1005–1015 (2002). 12235120 10.1083/jcb.200202051 PMC2173220

