# Supplementary material for: Force-transducing molecular ensembles at growing microtubule tips control mitotic spindle size
Source: Nat Commun. 2024 Nov 14;15:9865. doi: 10.1038/s41467-024-54123-2 (PMC11564643; doi:10.1038/s41467-024-54123-2)
Supplement: Supplementary file 1 — Supplementary Information [file 41467_2024_54123_MOESM1_ESM.pdf]

Supplementary Information

Force-transducing molecular ensembles at growing microtubule tips control mitotic spindle size

Authors

Lee-Ya Chu[1], Daniel Stedman[1,2], Julian Gannon[1], Susan Cox[2], Georgii Pobegalov[1,3] and Maxim I. Molodtsov[1,3,*]

Affiliations

[1] The Francis Crick Institute, London, NW1 1AT, United Kingdom

[2] King's College London, London, WC2R 2LS, UK

[3] Department of Physics and Astronomy, University College London, London, WC1E 6BT, United Kingdom

* **Correspondence**:

m.molodtsov@ucl.ac.uk

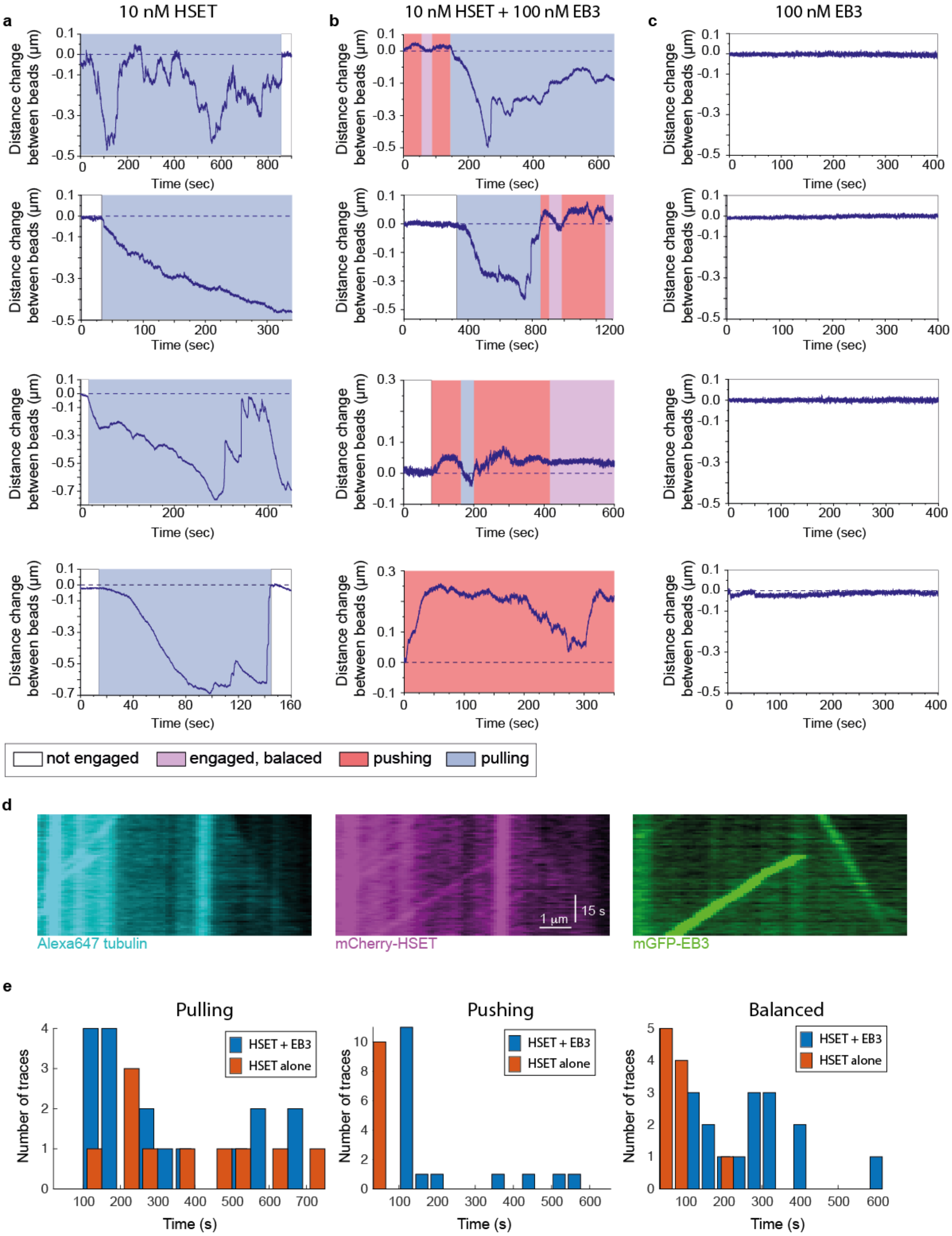

Supplementary Fig. 1 Additional examples and quantification of the double bead artificial spindle assay. a, Additional examples of traces in the presence of HSET alone. **b,** Additional examples of traces in the presence of HSET and EB. Colour shows where the system is experiencing pushing and pulling forces and where it is balanced. **c,** Examples of traces in the presence of EB alone. No force detected and asters do not visibly engage. In 'a-c' only the distance between the optically trapped beads is shown (force is linearly

proportional to the distance). **d,** Kymograph shows translocation of EB3 molecules by HSET towards microtubule minus ends indicating interaction between EB3 and HSET. **e,** Quantification of the distribution between the times the system spends on pulling, pushing and balanced states for all individual traces. Source data for this figure are provided as a Source Data file.

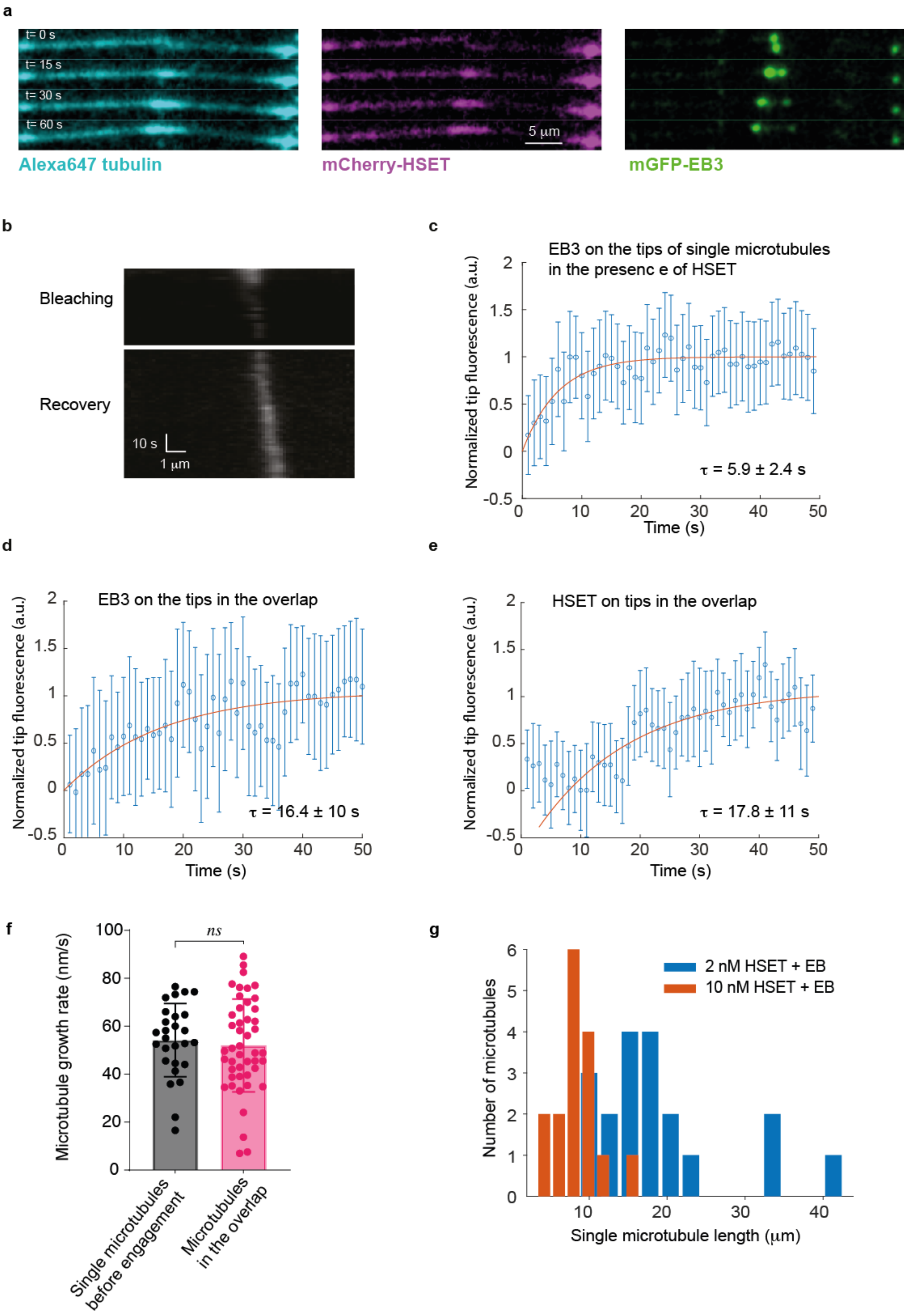

Supplementary Fig. 2 Quantification of HSET and EB3 dynamics in microtubule overlaps. a, Still images of individual channels are shown for the buckling microtubules

from example in Fig. 2a. **b,** Kymograph showing bleaching and photorecovery of EB3 on a growing microtubule tip. Bleaching and recovery parts of the kymograph were imaged at 100% and 20% of laser power respectively and are scaled to appear similar in brightness. **c,** Normalized and background subtracted fluorescence during recovery of EB3 following photobleaching on tips of single microtubules (not in overlaps), but in the presence of HSET. N=11 traces averaged. **d,** Normalized and background subtracted recovery of fluorescence after photobleaching of EB3 at the tips of microtubules engaged in antiparallel overlaps. N = 9 traces averaged. **e,** Normalized and background subtracted recovery of fluorescence after photobleaching of HSET at the tips of microtubules engaged in antiparallel overlaps. N = 9 traces averaged. In **c,-e,** errors for datapoints are SD between individual traces. Red lines are fits to $y(x) = a-b*exp(-x/\tau)$. Extracted value of τ from the fit is shown $\pm$ 95% confidence interval. a.u. stands for mean grey value corresponding to EMCCD camera counts. **f,** Comparison of the microtubule growth rates measured by tracking EB3 on single independent microtubules vs microtubules engaged in antiparallel overlaps. Bars show mean and whiskers standard deviation. N = 27, 46 (left to right). P-value is 0.52 by two-sided Wilcoxon test. **g,** Distribution of the single microtubule lengths used to extract values of force in Fig. 2f. Source data for this figure are provided as a Source Data file.

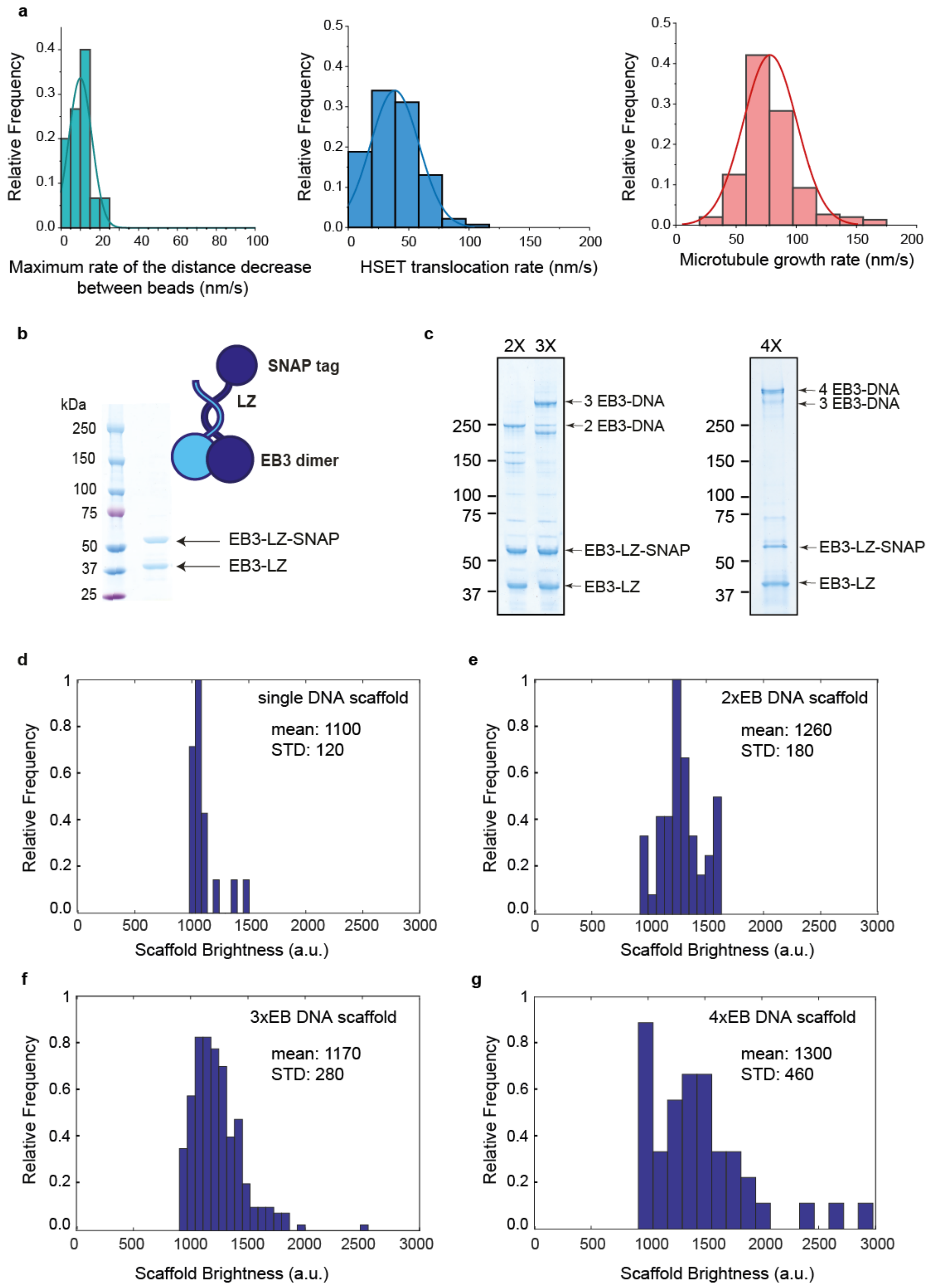

Supplementary Fig. 3 Characterization of the DNA scaffolds. a, Quantification of the rate of decrease between the two optically trapped bead driven by HSET action in the antiparallel microtubule overlaps (left, no EB present), speed of individual HSET translocation towards microtubule minus ends along individual microtubules measured in TIRF assay (middle), and individual microtubule growth rate in the buckling experiments (right). Left to right: n=20,

n=166, n=166. **b,** Denaturing SDS-PAGE of the purified EB3-LZ-SNAP/EB3-LZ heterodimers. The heterodimer with a single SNAP tag ensures that it can attach only to a single position of DNA backbone. **c,** SDS-PAGE (not boiled) of the EB3 ensembles coupled to DNA. 2x, 3x and 4x indicate samples with 2,3 and 4 EBs per scaffold correspondingly. SDS in the gel separates EB3 heterodimers. The sample was not heated, which prevented melting of the dsDNA structure and kept the scaffold intact. The major top bands in each case (arrows) correspond to scaffolds with the expected number of attached EBs. Gel quantification shows that the fraction of scaffolds with the expected number of EB3 dimers were 72% (2x), 55% (3x) and 86% (4x). **d,** Control experiment in which individual DNA scaffolds were adsorbed directly on the surface of the coverslip and brightness of the individual Cy3 dyes associated with single scaffolds were quantified. **e,** Brightness of 2xEB DNA scaffolds that were used to determine the run lengths of the 2xEB complexes on growing microtubule tips. Their brightness corresponds to the single Cy3 fluorophore from A. **f-g,** Same as in 'd', but for scaffolds with 3 or 4 EB dimers respectively. Mean and standard deviation numbers are shown for each distribution. a.u. stands for mean grey value corresponding to EMCCD counts. Source data for this figure are provided as a Source Data file.

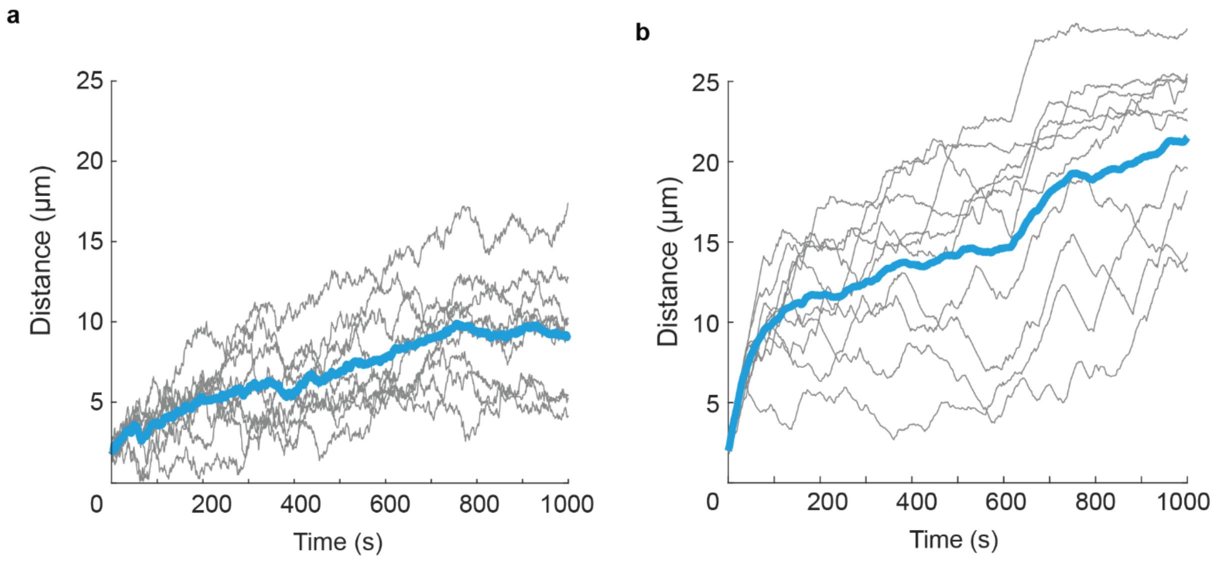

Supplementary Fig. 4 Additional simulations of the pole-to-pole distance between two asters of dynamic microtubules interacting via HSET and EB/HSET. a, Simulation in which total number of HSET and EB/HSET complexes was reduced from 1200 to 60. Individual traces experience more noise from increased stochasticity but the steady state distance between poles remains the same as in Fig. 4g indicating that it does not depend on the absolute number of motors. **b,** Simulation in which speed of microtubule growth was 4x higher than the speed of the HSET movement (250 nm/s and 60 nm/s respectively). Simulations show increased distance between poles due to longer microtubules and their faster growth rate. Source data for this figure are provided as a Source Data file.

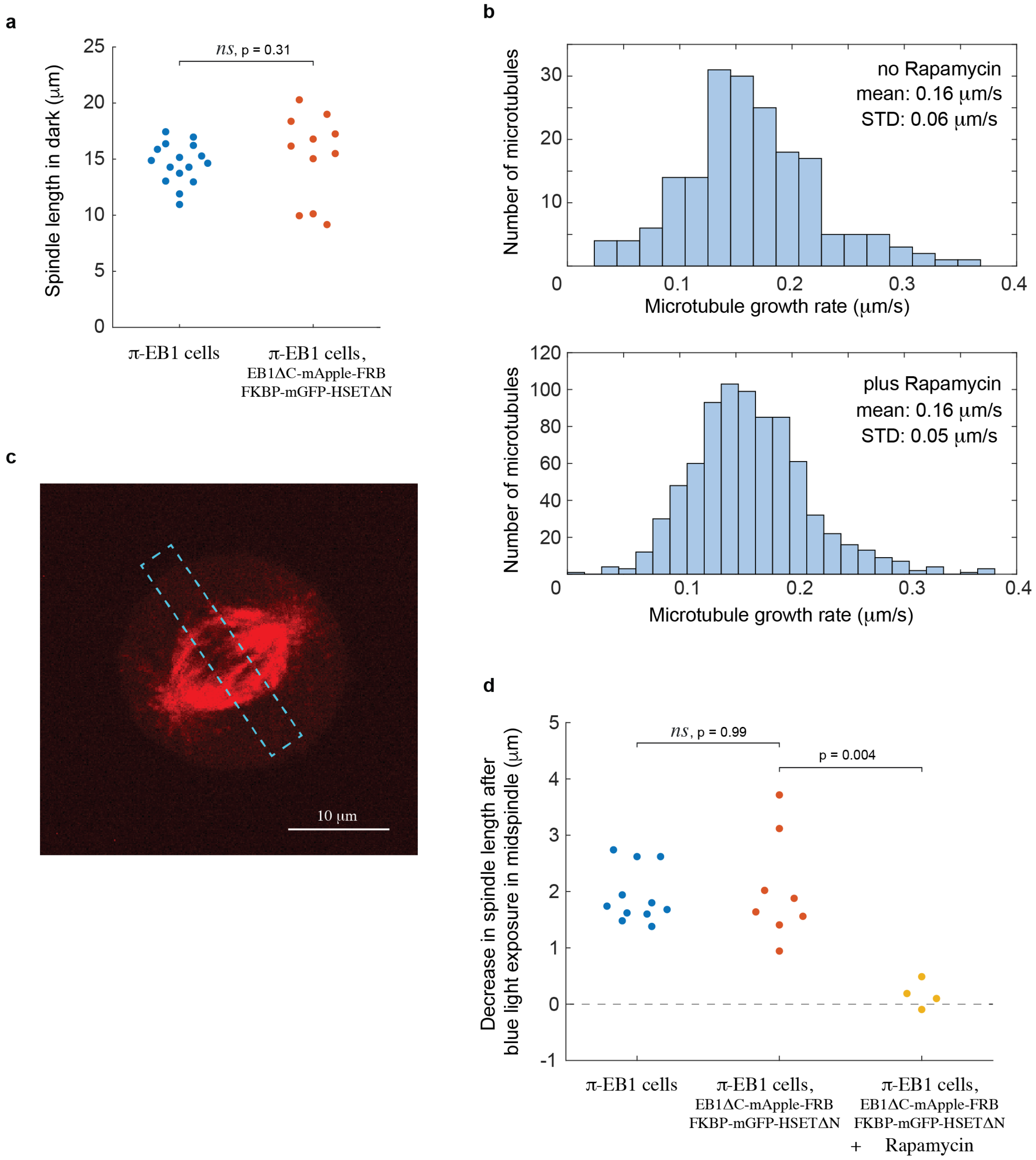

Supplementary Fig. 5 Additional quantification of optogenetic experiments. a, Comparison of the spindle lengths in the original π-EB1 H1299 cells (blue, data from Dema et al., 2022) and π-EB1 cells H1299 cells additionally expressing EB1ΔC-mApple-FRB and FKBP-mGFP-HSETΔN (red, this study). P-value is 0.31 by two-sided Wilcoxon test (n=16, 11)). **b,** Microtubule growth rate was measured in π-EB1 cells coexpressing EB1ΔC-mApple-FRB and FKBP-mGFP-HSETΔN in the absence and presence of rapamycin. n=185 (top) and n=783 (bottom) **c,** Image of the H1299 mitotic spindle cell in which EB1 was deactivated locally in the midzone spindle. Dotted line shows area of the optogenetic activation. **d,** Changes in the spindle length after deactivating EB1 in the midzone spindle. Conditions are shown on the x-axis. *ns* – not significant. P-values equal 0.99 and 0.004 by two-sided Wilcoxon test (n=11, 8, 4). Source data for this figure are provided as a Source Data file.

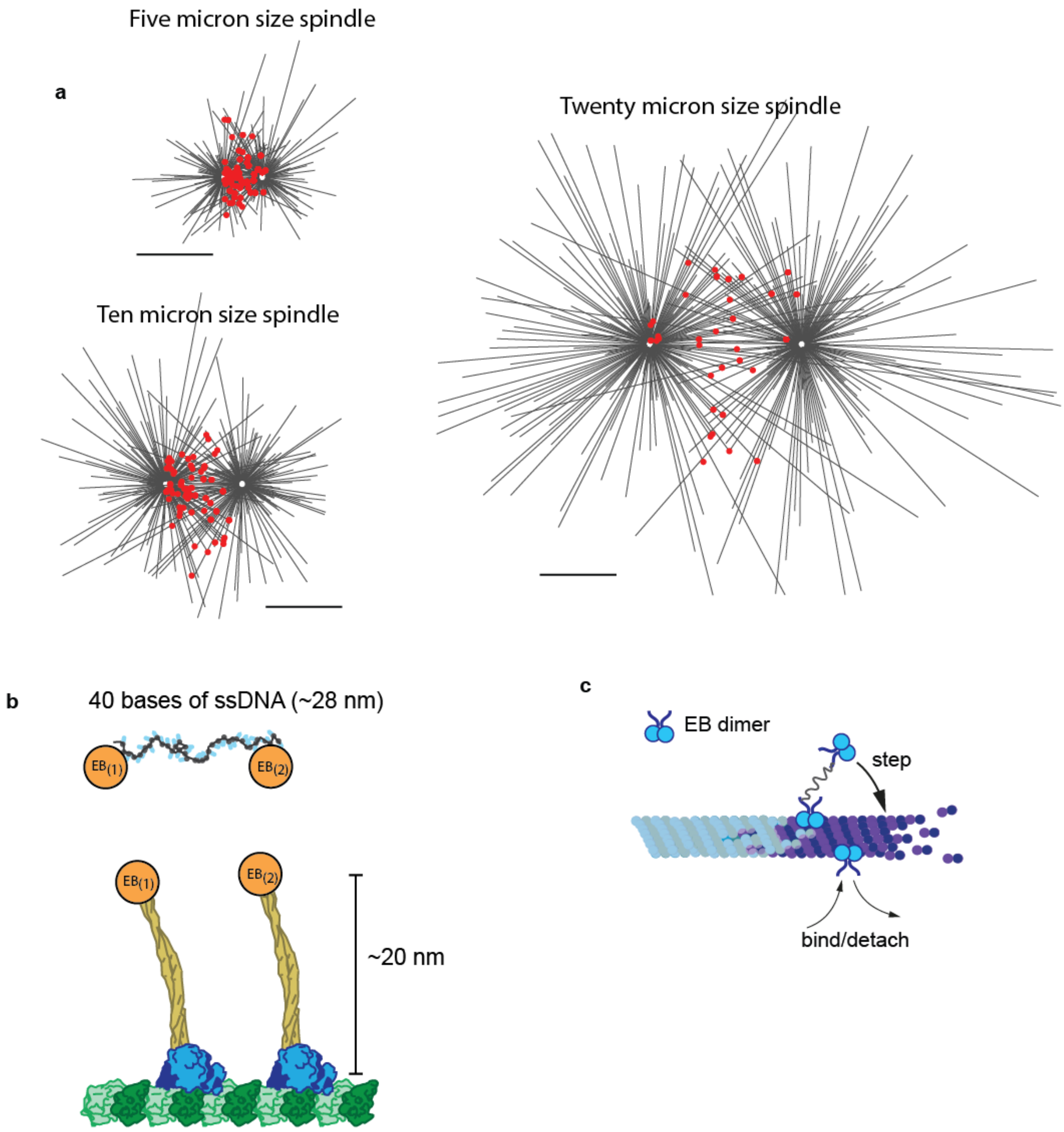

Supplementary Fig. 6 Geometry of the tip/lattice intersections and lattice/lattice overlaps, and EB scaffolds formed by interaction with HSET. a, Snapshots from simulations with five, ten and twenty micron-long spindle. Red dots highlight antiparallel tip/lattice intersections. Scale bars are 10 µm. **b,** Approximately to scale representation of two EB3 molecules coupled to a flexible ssDNA scaffold and two neighbouring EB3 binding sites on HSET molecules bound to microtubule lattice. The coiled-coil region of the HSET is ~ 20 nm. Distance between two HSET as shown is 16 nm (two tubulin dimers) but can be different depending on the HSET positions. **c,** Schematics showing how flexible connection between two microtubule tip-tracking dimers enables their processive movement with the growing microtubule tip.

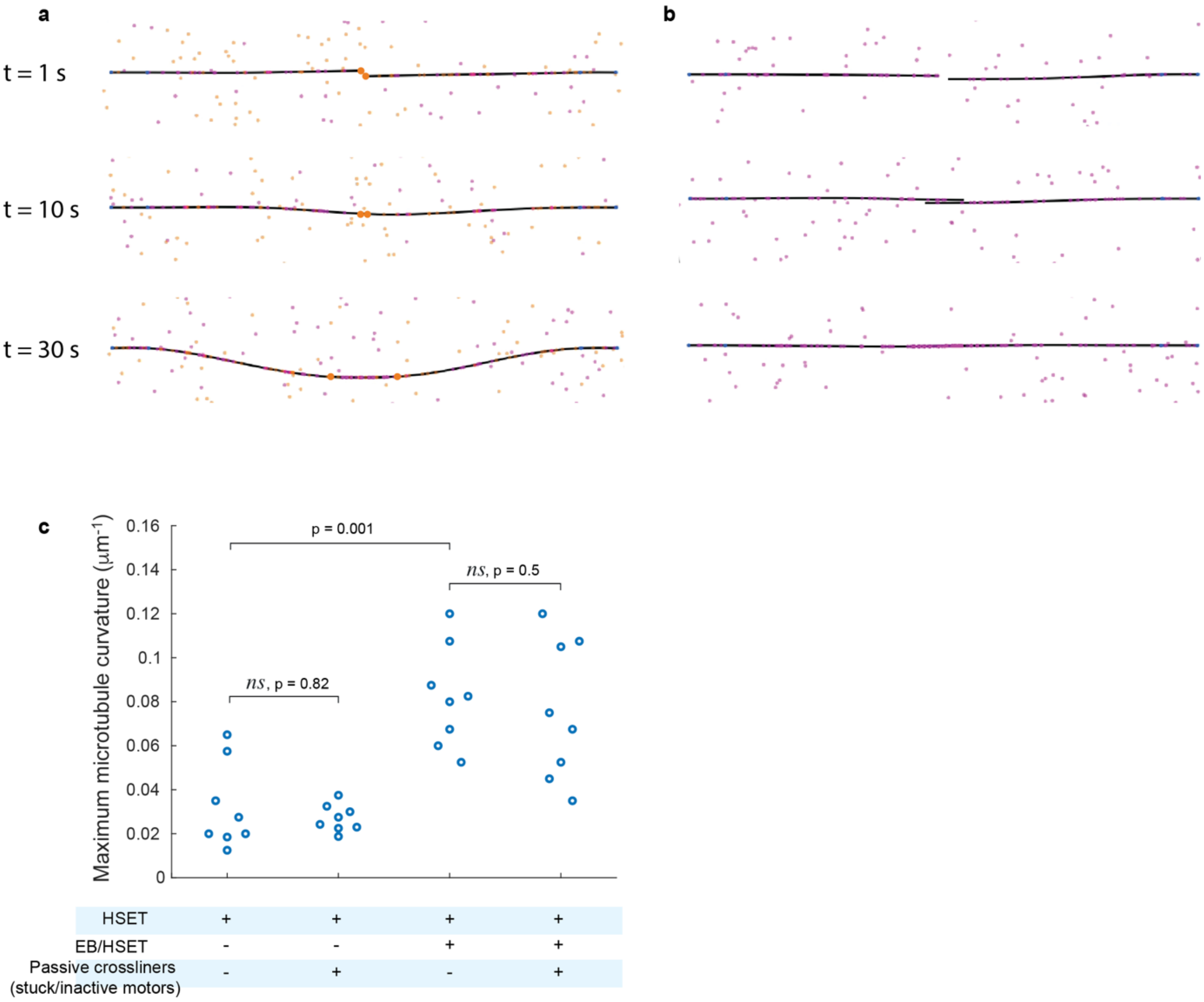

Supplementary Fig. 7 Cytosim simulations of two microtubules forming antiparallel overlap. a, Snapshots from a simulation with both HSET and EB3 molecules. **b,** Snapshots from a simulation with HSET only. EB3/HSET complexes are orange and HSET is magenta. **c,** Quantification of maximum microtubule curvatures following engagement in simulations in the presence of EB3, HSET and passive crosslinkers. Fraction of passive crosslinkers was 30%. Other parameters see in Methods. P-values are by two-sided Wilcoxon test (n=8 in each condition). Source data for this figure are provided as a Source Data file.

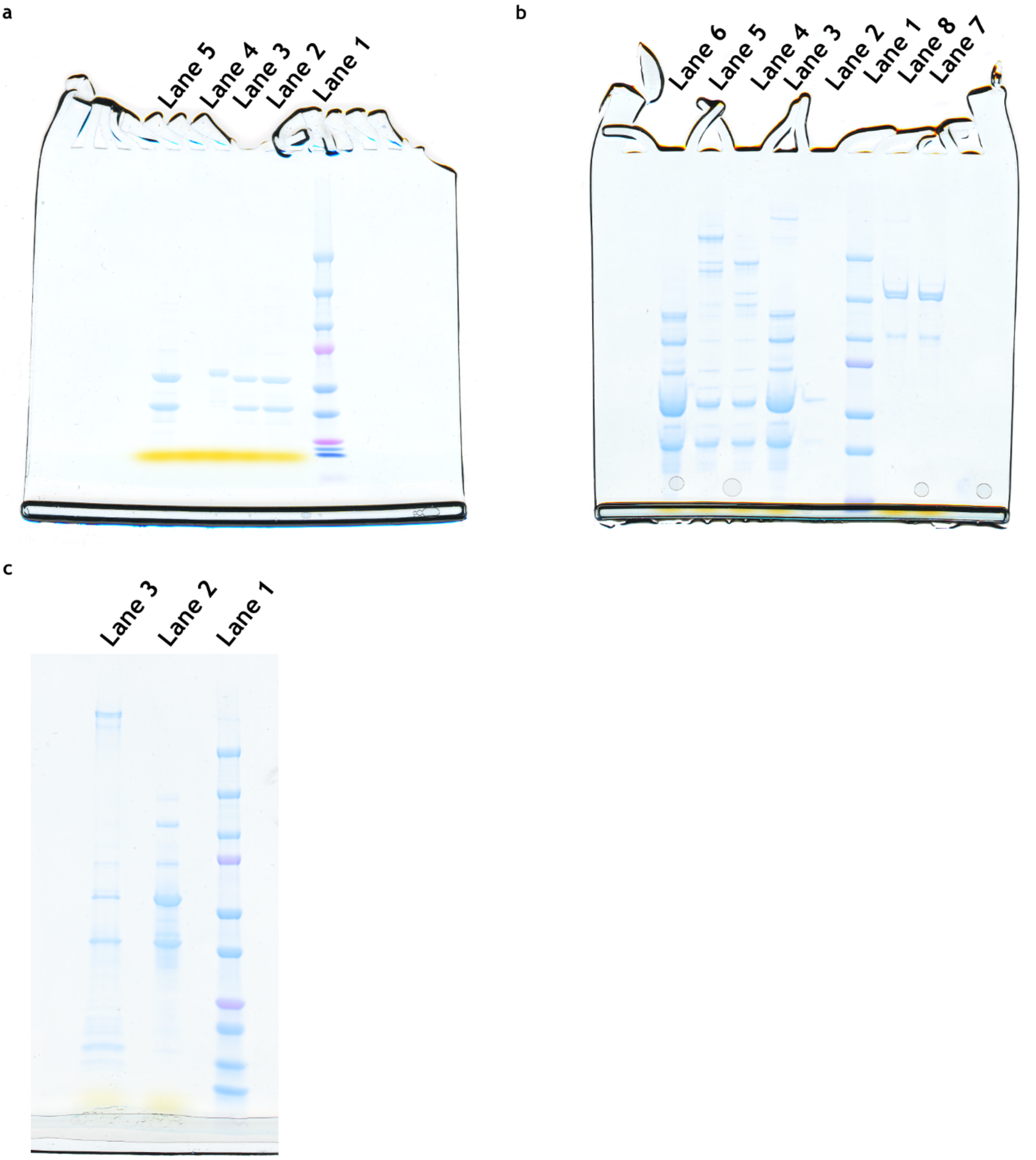

Supplementary Fig. 8 Uncropped scans of gels. a, Raw SDS-PAGE scan for Supplementary Fig. 3b. Lane 2 and Lane 3 showing the Coomassie blue stain of EB3-LZ-SNAP/EB3-LZ heterodimers. b, Raw SDS-PAGE scan for Supplementary Fig. 3c, 2x and 3x EB3 ensembles coupled to DNA (Lane 4 and Lane 5). c, Raw SDS-PAGE scan for Supplementary Fig. 3c, 4x EB3 ensembles coupled to DNA (Lane 3).
