# Supplementary material for: Force-transducing molecular ensembles at growing microtubule tips control mitotic spindle size
Source: Nat Commun. 2024 Nov 14;15:9865. doi: 10.1038/s41467-024-54123-2 (PMC11564643; doi:10.1038/s41467-024-54123-2)
Supplement: Supplementary file 2 — Description of Additional Supplementary Information [file 41467_2024_54123_MOESM2_ESM.docx]

**Description of Additional Supplementary Files**

File Name: Supplementary Movie 1

Description: Artificial spindle optical tweezers experiment with 10 nM HSET

File Name: Supplementary Movie 2

Description: HSET transports EB to the microtubule minus ends. HSET/EB3 interaction allows EB3 to be transported to the microtubule minus end by HSET. For the corresponding kymograph see Supplementary Fig. 1d.

File Name: Supplementary Movie 3

Description: Artificial spindle optical tweezers experiment with 10 nM HSET and 100 nM EB3. Movie showing crosslinking of antiparallel microtubules in the presence of EB3 and HSET. This movie corresponds to the generation of pushing force.

File Name: Supplementary Movie 4

Description: Two antiparallel microtubules buckle in the presence of EB3 and HSET. Related to Fig. 2a.

File Name: Supplementary Movie 5

Description: Simulation of the two-aster dynamics driven by HSET. In this simulation fraction of EB/HSET complexes to HSET alone was 10% and dynamics was dominated by the action of HSET. That led to the two asters fusion. Color legend: white – microtubules, magenta – unbound HSET, dark blue – unbound EB/HSET, pink – bound HSET, Light blue – bound EB/HSET.

File Name: Supplementary Movie 6

Description: Simulation of the two-aster dynamics driven by EB/HSET. In this simulation fraction of EB/HSET complexes in relation to HSET alone was 90%. In these case asters separate and stabilize in bipolar configuration. Colors follow Supplementary Movie 5.

File Name: Supplementary Movie 7

Description: Dissociation of π-EB1 from the microtubule plus-ends by blue-light stimulation in π-EB1 H1299 cell lines. Control experiment shows disappearance of EB1 comets after blue-light activation in interphase cells.

File Name: Supplementary Movie 8

Description: Active spindle shortening in π-EB1 H1299 cell lines without Rapamycin treatment after blue-light activation. EB1ΔC-mApple-FRB is in red, and FKBP-mGFP-HSETΔN is in green. Related to Fig. 5b.

File Name: Supplementary Movie 9

Description: Mitotic π-EB1 H1299 cell after rapamycin treatment and blue-light activation. EB1ΔC-mApple-FRB is in red, and FKBP-mGFP-HSETΔN is in green. Related to Fig. 5b.

File Name: Supplementary Movie 10

Description: Artificial spindle optical tweezers experiment with 10 nM HSET and higher density of microtubule seeds on the beads than in Supplementary Movie 1. Notations follow Supplementary Movie 1.

File Name: Supplementary Movie 11

Description: Simulation of the antiparallel overlap formation driven by EB/HSET. For parameters see Supplementary Fig. 7 and Methods. Color legend: white – microtubules, green – HSET, blue – EB/HSET. orange – microtubule anchors.

File Name: Supplementary Movie 12

Description: Simulation of the antiparallel overlap formation in the presence of HSET alone. For parameters see Supplementary Fig. 7 and Methods. Colors follow Supplementary Movie 11.
