# Supplementary material for: Force-transducing molecular ensembles at growing microtubule tips control mitotic spindle size
Source: Nat Commun. 2024 Nov 14;15:9865. doi: 10.1038/s41467-024-54123-2 (PMC11564643; doi:10.1038/s41467-024-54123-2)
Supplement: Supplementary file 15 — Reporting Summary [file 41467_2024_54123_MOESM15_ESM.pdf]

nature portfolio

Corresponding author(s): Maxim Molodtsov

Last updated by author(s): Oct 28, 2024

Reporting Summary

Nature Portfolio wishes to improve the reproducibility of the work that we publish. This form provides structure for consistency and transparency in reporting. For further information on Nature Portfolio policies, see our Editorial Policies and the Editorial Policy Checklist.

Statistics

For all statistical analyses, confirm that the following items are present in the figure legend, table legend, main text, or Methods section.

n/a	Confirmed	
☐	☒	The exact sample size (*n*) for each experimental group/condition, given as a discrete number and unit of measurement
☐	☒	A statement on whether measurements were taken from distinct samples or whether the same sample was measured repeatedly
☐	☒	The statistical test(s) used AND whether they are one- or two-sided<br>*Only common tests should be described solely by name; describe more complex techniques in the Methods section.*
☐	☒	A description of all covariates tested
☐	☒	A description of any assumptions or corrections, such as tests of normality and adjustment for multiple comparisons
☐	☒	A full description of the statistical parameters including central tendency (e.g. means) or other basic estimates (e.g. regression coefficient) AND variation (e.g. standard deviation) or associated estimates of uncertainty (e.g. confidence intervals)
☐	☒	For null hypothesis testing, the test statistic (e.g. *F*, *t*, *r*) with confidence intervals, effect sizes, degrees of freedom and *P* value noted<br>*Give P values as exact values whenever suitable.*
☒	☐	For Bayesian analysis, information on the choice of priors and Markov chain Monte Carlo settings
☒	☐	For hierarchical and complex designs, identification of the appropriate level for tests and full reporting of outcomes
☒	☐	Estimates of effect sizes (e.g. Cohen's *d*, Pearson's *r*), indicating how they were calculated

Our web collection on statistics for biologists contains articles on many of the points above.

Software and code

Policy information about availability of computer code

Data collection	The JPK optical trap control software (v 6.1) was employed to operate the optical trap and collect data for the single-beam optical experiments.<br>The Andor Solis (v 4.31) software was used to acquire and record microscopy data simultaneous with force application/measurements by the JPK software.<br>Lumicks C-trap control software is BlueLake (v1.6)
Data analysis	The JPK Processing (v 6.1) Software was used to extract force traces in the single-beam optical trapping data.<br>Python (v3.9) was used for the analysis of the double bead C-trap data.<br>FIJI (ImageJ, v 1.54) was used for visualisation and image analysis.<br>Custom-made MATLAB (R2022b) codes were used to for single-molecule fluorescence intensity analysis<br>Force determination and visualisation, histogram fitting, statistical testing and simulations.<br>For custom codes see: https://github.com/FrancisCrickInstitute/MT_Force_Spindle.

For manuscripts utilizing custom algorithms or software that are central to the research but not yet described in published literature, software must be made available to editors and reviewers. We strongly encourage code deposition in a community repository (e.g. GitHub). See the Nature Portfolio guidelines for submitting code & software for further information.

Data

Policy information about availability of data

All manuscripts must include a data availability statement. This statement should provide the following information, where applicable:

- Accession codes, unique identifiers, or web links for publicly available datasets
- A description of any restrictions on data availability
- For clinical datasets or third party data, please ensure that the statement adheres to our policy

This paper does not contain any data with mandatory deposition. Source data used for the generation of figures is provided with this paper. The rest of the raw data is available by the authors upon request. Software used for analysis and simulations are publicly available on GitHub: https://github.com/FrancisCrickInstitute/MT_Force_Spindle.

Research involving human participants, their data, or biological material

Policy information about studies with human participants or human data. See also policy information about sex, gender (identity/presentation), and sexual orientation and race, ethnicity and racism.

Reporting on sex and gender	N/A
Reporting on race, ethnicity, or other socially relevant groupings	N/A
Population characteristics	N/A
Recruitment	N/A
Ethics oversight	N/A

Note that full information on the approval of the study protocol must also be provided in the manuscript.

Field-specific reporting

Please select the one below that is the best fit for your research. If you are not sure, read the appropriate sections before making your selection.

[x] Life sciences [ ] Behavioural & social sciences [ ] Ecological, evolutionary & environmental sciences

For a reference copy of the document with all sections, see nature.com/documents/nr-reporting-summary-flat.pdf

Life sciences study design

All studies must disclose on these points even when the disclosure is negative.

Sample size	The sample size was not predetermined. Data was collected until the statistical significance was reached.
Data exclusions	We did not exclude any data.
Replication	The number of repeat measurements made is stated in figure legends. All experiments were repeated at least three times, performed on different days and in independent flow cells with fresh proteins.
Randomization	Single-molecule data with the same conditions were inherently randomized and no other special randomization was implemented.
Blinding	Investigators were not blinded. All samples were prepared on the same day as the measurement were performed by the same person.

Reporting for specific materials, systems and methods

We require information from authors about some types of materials, experimental systems and methods used in many studies. Here, indicate whether each material, system or method listed is relevant to your study. If you are not sure if a list item applies to your research, read the appropriate section before selecting a response.

Materials & experimental systems

n/a	Involved in the study
☐	☒ Antibodies
☐	☒ Eukaryotic cell lines
☒	☐ Palaeontology and archaeology
☒	☐ Animals and other organisms
☒	☐ Clinical data
☒	☐ Dual use research of concern
☒	☐ Plants

Methods

n/a	Involved in the study
☒	☐ ChIP-seq
☒	☐ Flow cytometry
☒	☐ MRI-based neuroimaging

Antibodies

Antibodies used	Anti-Digoxigenin-AP, Fab fragments. Roche, 11093274910.
Validation	This commercially available antibody was validated by the manufacturer, Roche, for the detection of digoxigenin-labeled compounds, such as Digoxigenin-labeled nucleic acids. In this work, antibodies were absorbed on the surface of a microfluidic flow-cell for the attachment of GMpCpp stabilized microtubule seeds labelled with Digoxygenin. The attachment was confirmed by imaging immobilized microtubule seeds using fluorescence microscopy. This experiment provided the required validation.

Eukaryotic cell lines

Policy information about cell lines and Sex and Gender in Research

Cell line source(s)	NCI-H1299 (ATCC)
Authentication	STR profiling
Mycoplasma contamination	Cells regularly tested negative for mycoplasma.
Commonly misidentified lines (See ICLAC register)	No cell lines used in this study were found in the database of commonly misidentified cell lines that is maintained by ICLAC and NCBI Biosample.

Plants

Seed stocks	N/A
Novel plant genotypes	N/A
Authentication	N/A
