# Supplementary material for: Force-transducing molecular ensembles at growing microtubule tips control mitotic spindle size
Source: Nat Commun. 2024 Nov 14;15:9865. doi: 10.1038/s41467-024-54123-2 (PMC11564643; doi:10.1038/s41467-024-54123-2)
Supplement: Supplementary file 16 — Transparent Peer Review file [file 41467_2024_54123_MOESM16_ESM.pdf]

nature portfolio

Peer Review File

Force-transducing molecular ensembles at growing microtubule tips control mitotic spindle size

Corresponding Author: Dr Maxim Molodtsov

This manuscript has been previously reviewed at another journal. This document only contains information relating to versions considered at Nature Communications.

This file contains all reviewer reports in order by version, followed by all author rebuttals in order by version.

Version 0:

Reviewer comments:

Reviewer #1

(Remarks to the Author)
The authors have revised their text and clarified my queries satisfactorily.
Without scalebar details (eg fig 5B), the images are difficult to interpret.
I would recommend adding scale bars to supplementary movies as well.

In movies, the time-lapse period needs to be indicated (unclear if the movies are minutes or seconds long).

Reviewer #3

(Remarks to the Author)

Open Access This Peer Review File is licensed under a Creative Commons Attribution 4.0 International License, which permits use, sharing, adaptation, distribution and reproduction in any medium or format, as long as you give appropriate credit to the original author(s) and the source, provide a link to the Creative Commons license, and indicate if changes were made.
In cases where reviewers are anonymous, credit should be given to 'Anonymous Referee' and the source.
The images or other third party material in this Peer Review File are included in the article's Creative Commons license, unless indicated otherwise in a credit line to the material. If material is not included in the article's Creative Commons license and your intended use is not permitted by statutory regulation or exceeds the permitted use, you will need to obtain permission directly from the copyright holder.
To view a copy of this license, visit https://creativecommons.org/licenses/by/4.0/
